# An explicitly designed paratope of amyloid-β prevents neuronal apoptosis *in vitro* and hippocampal damage in rat brain†

**DOI:** 10.1039/d0sc04379f

**Published:** 2020-12-22

**Authors:** Ashim Paul, Sourav Kumar, Sujan Kalita, Sourav Kalita, Dibakar Sarkar, Anirban Bhunia, Anupam Bandyopadhyay, Amal Chandra Mondal, Bhubaneswar Mandal

**Affiliations:** Laboratory of Peptide and Amyloid Research, Department of Chemistry, Indian Institute of Technology Guwahati (IITG) North Guwahati Assam-781039 India bmandal@iitg.ac.in; Neuroscience Research Unit, Department of Physiology, Raja Peary Mohan College Hooghly Uttarpara West Bengal-712258 India acmondal@mail.jnu.ac.in; Biomolecular NMR and Drug Design Laboratory, Department of Biophysics, Bose Institute P-1/12 CIT Scheme VII (M) Kolkata 700054 India; Peptide Engineering Laboratory, Department of Chemistry, Indian Institute of Technology Ropar Punjab-140001 India; Laboratory of Cellular & Molecular Neurobiology, School of Life Sciences, Jawaharlal Nehru University New Delhi-110 067 India

## Abstract

Synthetic antibodies hold great promise in combating diseases, diagnosis, and a wide range of biomedical applications. However, designing a therapeutically amenable, synthetic antibody that can arrest the aggregation of amyloid-β (Aβ) remains challenging. Here, we report a flexible, hairpin-like synthetic paratope (SP1, ∼2 kDa), which prevents the aggregation of Aβ monomers and reverses the preformed amyloid fibril to a non-toxic species. Structural and biophysical studies further allowed dissecting the mode and affinity of molecular recognition events between SP1 and Aβ. Subsequently, SP1 reduces Aβ-induced neurotoxicity, neuronal apoptosis, and ROS-mediated oxidative damage in human neuroblastoma cells (SH-SY5Y). The non-toxic nature of SP1 and its ability to ameliorate hippocampal neurodegeneration in a rat model of AD demonstrate its therapeutic potential. This paratope engineering module could readily implement discoveries of cost-effective molecular probes to nurture the basic principles of protein misfolding, thus combating related diseases.

## Abstract 2

Herein, the therapeutic potentials of an explicitly designed peptide probe are systematically illuminated *in vitro* and *in vivo* against Aβ aggregation. The probe demonstrates remarkable potency for attenuating neurotoxicity and hippocampal damage.

## Graphical abstract

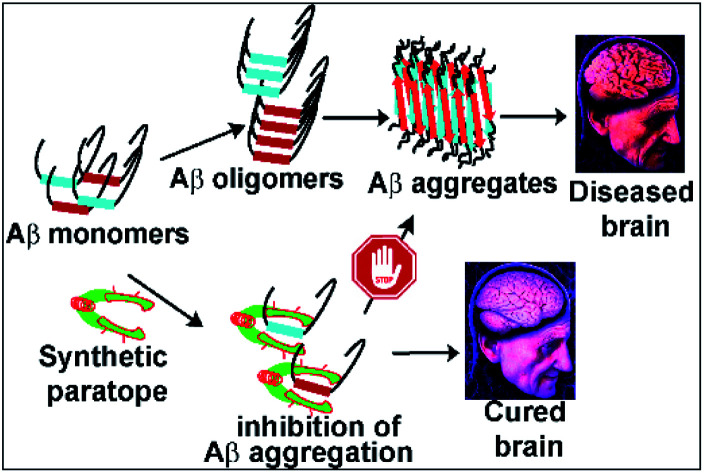

## Introduction

The deposition of amyloid fibrils has consequences with numerous protein-misfolding diseases, including Alzheimer's, Parkinson's, and Huntington's disease, Prion diseases, and type-2 diabetes.^1,2^ The detailed molecular mechanism of Alzheimer's disease (AD) is not intelligible yet. However, growing shreds of evidence suggest that the aggregation of amyloid-β peptide (Aβ) from native non-toxic monomers to highly toxic amyloid fibrils in the extracellular space and formation of neurofibrillary tangles (NFTs) in neurons are the principal hallmarks for the pathogenesis of AD.^3,4^ In the past two decades, numerous strategies have been exercised to find a cure for AD.^5^ These strategies involve metal chelators, nanoparticles, the amyloidogenic core region (KLVFF)^6–8^ or other fragments of the Aβ peptide,^9,10^ chemical chaperones,^11,12^ peptide-based inhibitors,^13–16^ small molecules,^5,17^ and conformation-selective antibodies.^18–21^ Antibody-based drug design is the most intriguing as antibodies engulf and eliminate the toxic Aβ species. Besides, antibodies have demonstrated the scope and potential of immunotherapy. Nevertheless, they are associated with severe adverse effects such as Fc-mediated pro-inflammatory immune responses. Recently, affibodies^22–26^ have shown prevention of the self-aggregation of Aβ by encapsulating the Aβ peptide and reducing pro-inflammatory immune responses, which led to a novel therapeutic approach against AD.^18–27^ Among the mentioned strategies, a rationally designed, short peptide from a self-aggregation site of Aβ showed promising results even in clinical trials with superior bioavailability and less toxicity.^5,28^

**Fig. 1:**
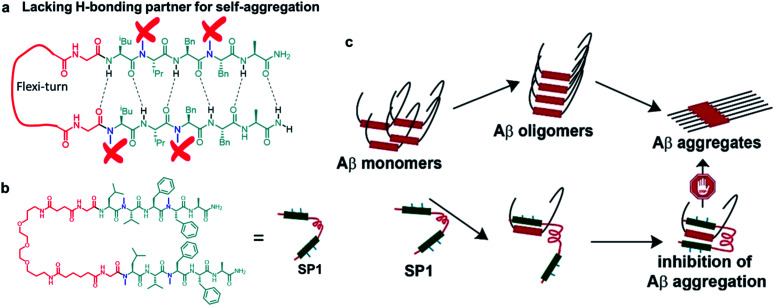
(a) Cartoon representation of the explicitly designed and assembled synthetic paratope (SP1). (b) The complete chemical structure of SP1. (c) Plausible mode of inhibition of aggregation by sequestration of Aβ monomers by SP1.

Here, we aimed to construct an explicitly designed synthetic paratope inspired by a peptide fragment of Aβ that could potentially be a clinical candidate for targeting Aβ. A paratope is a part of an antibody known to recognize the epitope region of an antigen selectively.^18–21^ The knowledge from prior investigations by our group and numerous reports has empowered us to construct a flexible, parallel β-hairpin-like synthetic paratope (SP1, Fig. 1a and b). The size of the designed SP1 is smaller than that of any existing antibody and affibody. We explored its efficiency in binding to Aβ using various spectroscopic techniques. The atomic-scale mechanistic study by NMR dissected the recognition mechanism. We show that SP1 remarkably disaggregates the preformed Aβ aggregates and potentially dissolves Aβ plaques through different *in vitro* studies. Besides, SP1 reduces Aβ_40_ induced cytotoxicity, oxidative stress-mediated apoptotic events, and dysregulation of Ca^2+^ homeostasis in human neuroblastoma SH-SY5Y cells.^29,30^SP1 also improves Aβ_40_ induced ROS generation and modulates apoptosis signalling in the cells. Notably, SP1 has therapeutic potential *in vivo* through less toxicity and ameliorating hippocampal neurodegeneration.

## Results and discussion

### Design and synthesis of the paratope

**Fig. 2:**
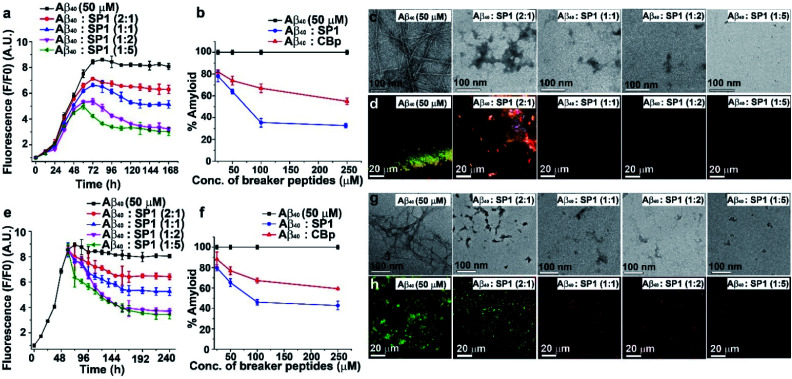
Modulation of Aβ_40_ aggregation: (a) time-dependent ThT assay of Aβ_40_ in the absence (black) or presence of different doses of SP1. (b) Dose-dependent ThT assay for the inhibition of amyloid formation by Aβ_40_ in the absence (black) or presence of SP1 (blue) and CBp (red). (c) TEM and (d) Congo red birefringence images of Aβ_40_ in the absence or presence of varying doses of SP1. (e) Time-dependent ThT assay for the disaggregation of preformed Aβ_40_ aggregates in the absence (black) or presence of different doses of SP1. (f) Dose-dependent ThT assay for the disaggregation of preformed Aβ_40_ aggregates in the absence (black) or presence of SP1 (blue) and CBp (red). (g) TEM and (h) Congo red birefringence images of Aβ_40_ in the absence or presence of varying doses of SP1.

**Fig. 3:**
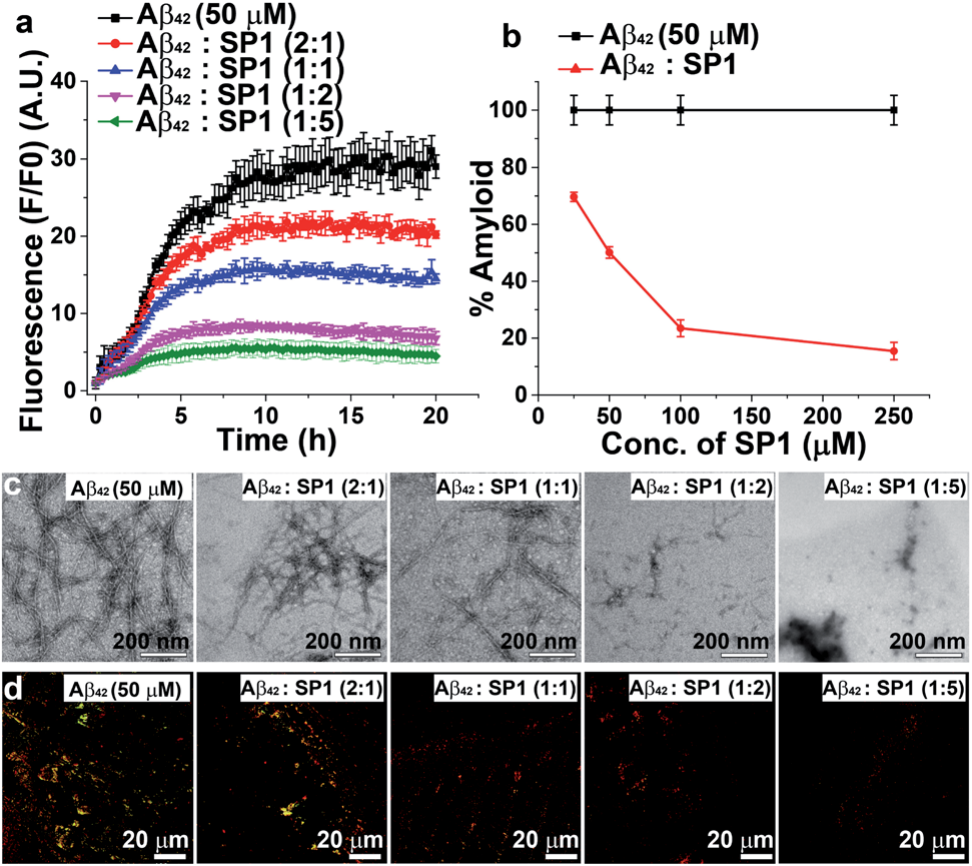
Inhibition of Aβ_42_ aggregation: (a) time-dependent ThT assay of Aβ_42_ in the absence (black) or presence of different doses of SP1. (b) Dose-dependent ThT assay for the inhibition of Aβ_42_ aggregate formation; absence of SP1 (black) or presence of SP1 (red). (c) TEM and (d) Congo red birefringence images of Aβ_42_ in the absence or presence of different doses of SP1.

**Fig. 4:**
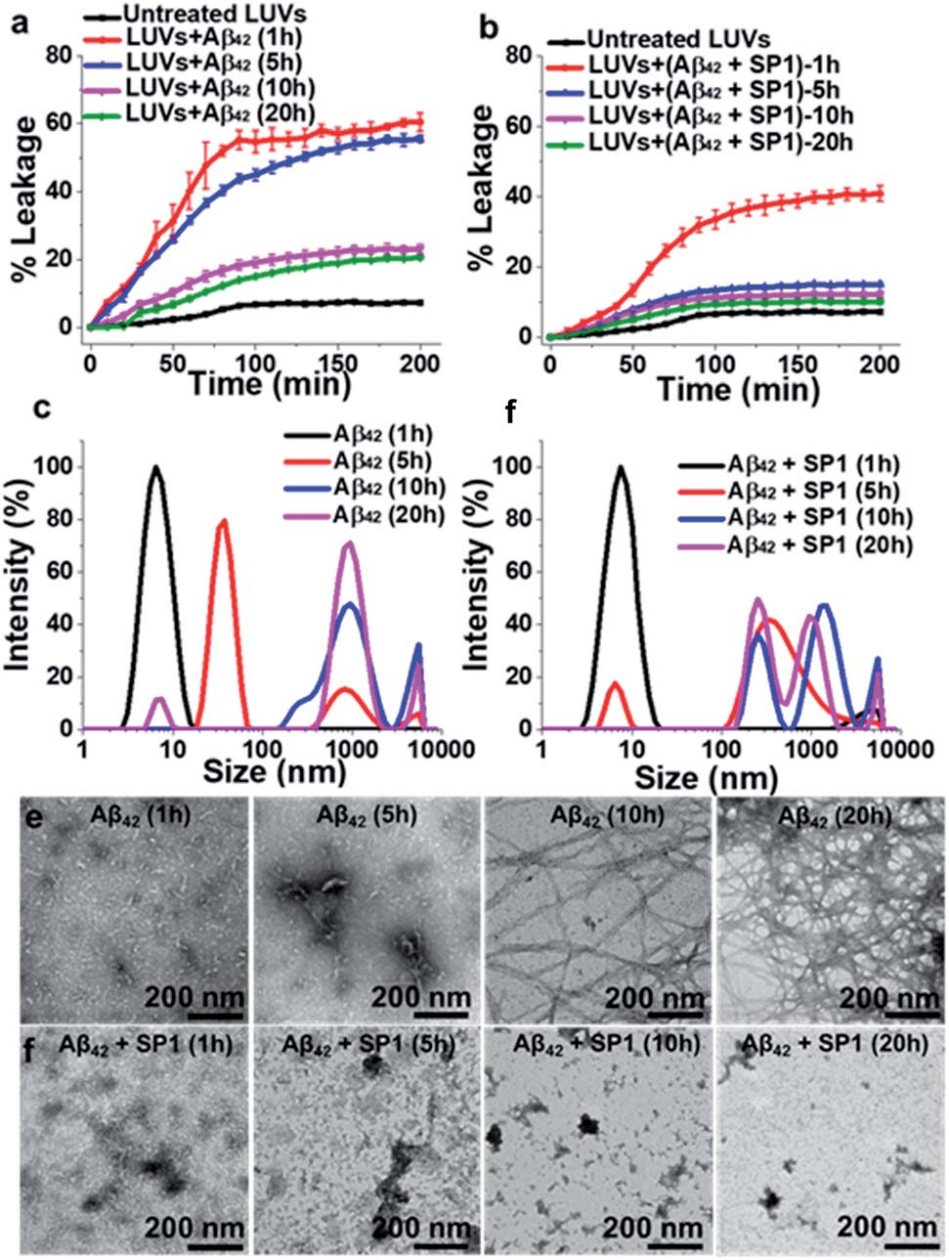
Reduction of Aβ_42_ oligomerization: (a and b) the effect of SP1 on LUV dye leakage caused by Aβ_42_ aggregates (incubated at varied time intervals: 1 h, 5 h, 10 h and 20 h) in the absence (a) or presence (b) of SP1 monitored by carboxyfluorescein dye emission. Spontaneous dye leakage from the LUVs is indicated as black curves. Complete dye leakage (100%) was achieved by treating LUVs with 10% Triton X-100. (c and d) The effect of SP1 on the size distribution of Aβ_42_ aggregates (incubated for varied time intervals) in the absence (c) or presence (d) of SP1 monitored by DLS. (e and f) The effect of SP1 on the morphology of Aβ_42_ aggregates (incubated for varied time intervals) in the absence (e) or presence (f) of SP1 monitored by TEM.

**Fig. 5:**
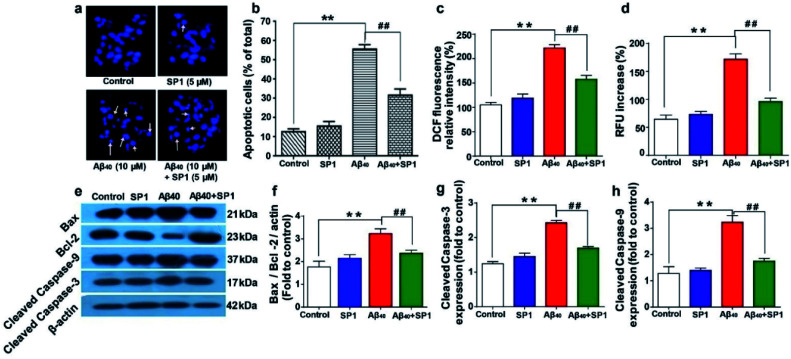
Amelioration of Aβ_40_ induced oxidative stress, cellular apoptosis, and Ca^2+^ dyshomeostasis by SP1: (a) fluorescence images of SH-SY5Y cells exposed to Aβ_40_ or SP1 alone and co-incubated with both of them for 24 h. The arrow indicates the condensed and fragmented nuclei. (b) Apoptotic cells with the altered nuclear structure were quantified after Aβ_40_ treatment in the presence or absence of SP1. (c) Effect of SP1 on Aβ_40_ induced ROS generation in SH-SY5Y cells after 24 h of incubation. The amount of intracellular ROS formation was determined by the oxidation of DCFH-DA to DCF. (d) Effect of SP1 on Aβ_40_ induced elevation of intracellular calcium (Ca^2+^) in SH-SY5Y cells. Effect of SP1 on Aβ_40_ (10 μM) induced Bcl-2, Bax, cleaved caspase-9, and cleaved caspase-3 expression in SH-SY5Y cells. (e) Representative images of western blot analysis of Bcl-2, Bax, cleaved caspase-9, and cleaved caspase-3 protein expression (for original blot, see ESI Table 2†). (f–h) Densitometric analysis of changes in levels of the Bcl-2/Bax expression ratio, cleaved caspase-9, and cleaved caspase-3 (changes fold to control), respectively. The protein bands were quantified using smart view image analysis, and values are expressed as mean ± SEM (*n* = 3 experiments per group) **p < 0.01, compared to the control group and ##*p* < 0.01 compared to Aβ_40_ treated group.

**Fig. 6:**
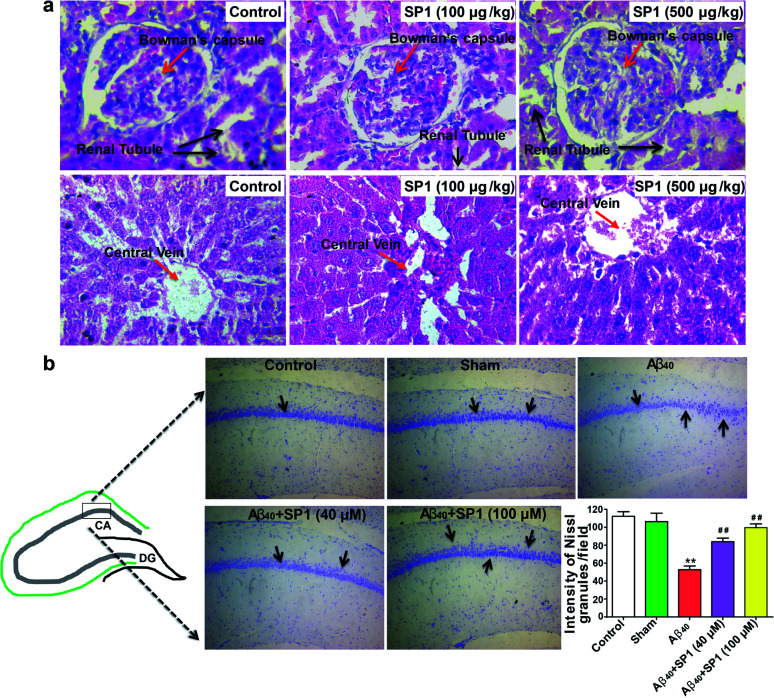
Amelioration of Aβ_40_-induced hippocampal neurodegeneration by SP1 in a rat model of AD: (a) effect of SP1 on histopathological studies on the liver and kidney for sub-chronic toxicity in rats (*n* = 8 each group). The image is shown at 40× magnification (haematoxylin and eosin stain). (b) Nissl granules of hippocampal neurons were observed by cresyl violet staining in the different experimental groups of rats (*n* = 6). There was a significant decrease in the intensity of Nissl granules in Aβ_40_-infused rats as compared to the control and the sham groups of rats. SP1 injected rats had a significant increase in the intensity of Nissl granules in the hippocampus when compared to only Aβ_40_-infused rats.

**Fig. 7:**
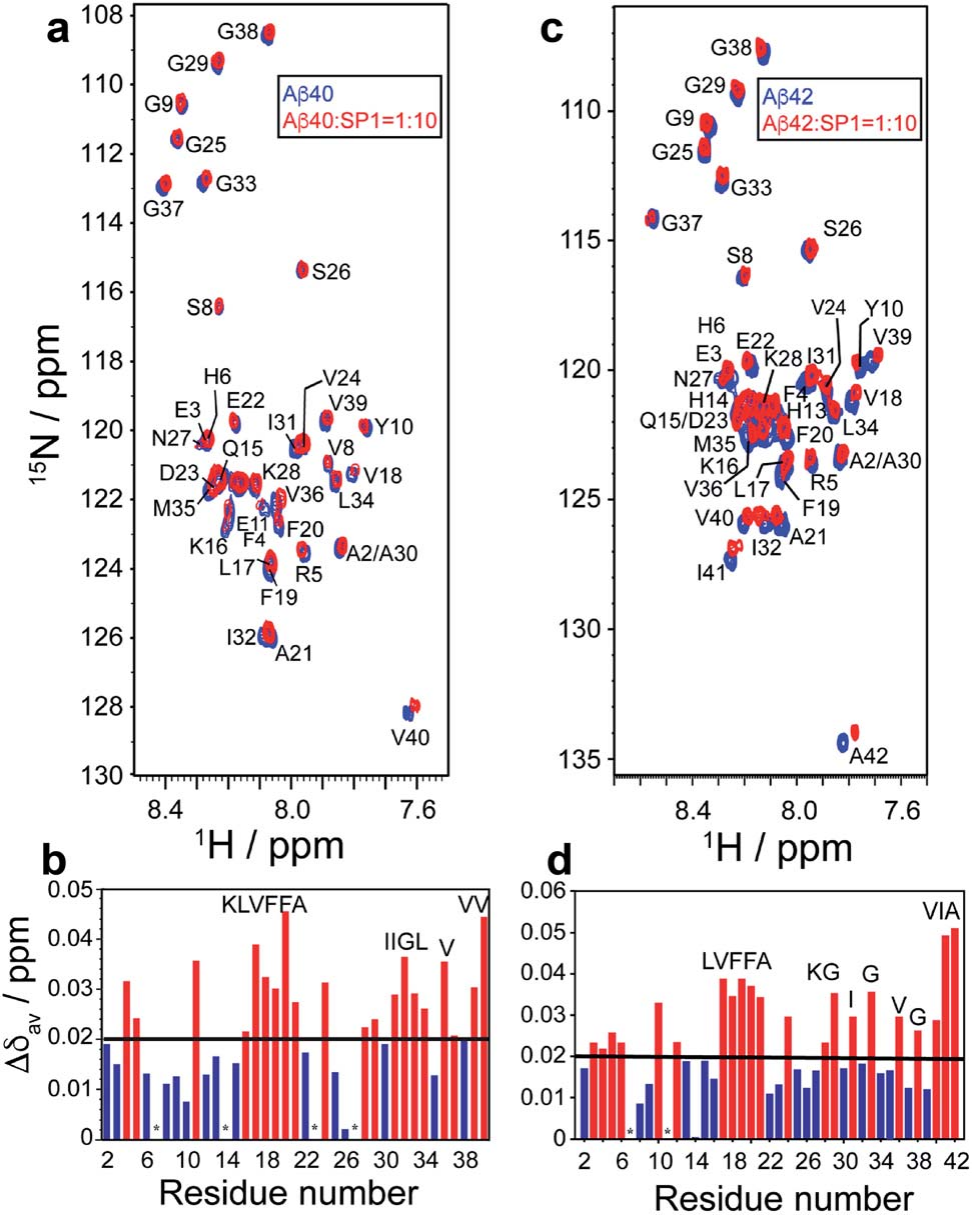
Molecular interaction between Aβ_40_/Aβ_42_ and SP1: (a and c) 2D 1H/15N-SOFAST HMQC spectra (Bruker Avance III 500 MHz NMR spectrometer, equipped with SMART probe, at 283 K) of 15N labeled (a) Aβ_40_ (80 μM) or (c) Aβ_42_ (80 μM), before (blue) and after (red) addition of SP1 at a 1 : 10 molar ratio. (b and d) Bar plot of CSP (Aβ_40_/Aβ_42_ : SP1 = 1 : 10) Aβ_40_/Aβ_42_ due to binding of SP1. The black threshold line represents the mean of all CSP values. Red bars indicate the most interacting residues. Overlapping residues with similar chemical shifts are marked with an asterisk (*).

The π → π stacking interactions play a central role in the self-assembly processes in most amyloidogenic proteins leading to their aggregation and disease progression.^31,32^ The central core hydrophobic region of the Aβ peptide (LVFFA), which acts as a self-recognition unit, was chosen as a strand in the designed hairpin-like SP1. Two strands were connected in parallel through a flexible unit (PEG) to construct the complete synthetic paratope molecule (Fig. 1a, b and ESI Scheme 1†). We introduced *N*-methylation^33,34^ in the alternate amino acids in each strand, preventing self-aggregation by blocking intermolecular H-bonding (Fig. 1a). In a similar principle, *N*-methylation should not allow further aggregation of the Aβ peptide captured by SP1. Since the central core hydrophobic region (epitope)^6–8^ of Aβ is crucial for self-aggregation and senile plaque formation, we designed SP1 in such a way that it can selectively bind with the epitope and capture Aβ from both sides with its two strands, as proposed in Fig. 1c. We introduced a control β-breaker peptide (CBp) with only one *N*-methylated strand of SP1. Also, five more peptides conjugated with suitable fluorophores were hosted to investigate the mechanism of interaction between SP1 and Aβ_40_ (ESI Table 1 and Fig. 1–7†). In the beginning, we confirmed that SP1 and CBp are non-amyloidogenic using combined CD, FTIR, TEM, and birefringence analyses (ESI Fig. 8 and 9†).

### Inhibition of Aβ_40_ amyloid formation

To investigate the inhibitory effect of SP1 on Aβ_40_ fibrillization, we performed various biophysical assays in the presence of different doses (0.5, 1, 2, and 5-fold molar excess of SP1), and CBp was used as a control. First, we monitored the kinetics of the amyloid formation of Aβ_40_ (50 μM) by thioflavin T (ThT) assay (Fig. 2a and b). The Aβ_40_ peptide alone aggregated with time, with a growth saturation point around 72 h, as evident from the surge in ThT fluorescence intensity. However, in the presence of SP1, the intensity of fluorescence decreased in a dose-dependent manner (Fig. 2a), and precisely, a 5-fold molar excess instigated ∼66% inhibition (Fig. 2b). Likewise, SP1 disaggregated the characteristic fibrillar aggregates of Aβ_40_ under TEM (Fig. 2c) in a dose-dependent manner. We inspected a few fibrillar aggregates at the lowest dose of SP1, demonstrating the essential requirement of an optimal concentration for Aβ_40_ disaggregation. Green-gold birefringence appears to be a standard result of Aβ_40_ aggregation under cross-polarized light post-staining with Congo red dye. Owing to amyloid formation, Aβ_40_ appears as green-gold birefringence (Fig. 2d) under cross-polarized light when stained with Congo red. Upon treatment with various doses of SP1, such type of birefringence (Fig. 2d) disappeared, except at the lowest dose. In contrast, the control peptide (CBp) showed ∼45% inhibition of Aβ_40_ peptide aggregation with the experiments mentioned above in parallel (ESI Fig. 10a–c†). We noticed that a 5-fold dose is the minimal requirement for CBp to inhibit aggregation, whereas equimolar SP1 completely prevents it.

### Disruption of the preformed Aβ_40_ aggregates

Understanding the kinetics of fibrillization led us to design an *in vitro* experiment of preformed fibril disaggregation. In this experiment, the 60 h aged Aβ_40_ peptide was incubated further for 180 h (total 240 h) with SP1 and CBp separately at varying doses (0.5, 1, 2, and 5-fold molar excess). The high fluorescence intensity of ThT shows that preformed Aβ_40_ fibrils were suppressed substantially with increased doses of SP1 and the control peptide (Fig. 2e and ESI Fig. 11a†). Distinctly, we observed ∼57% (Fig. 2f) and ∼39% (Fig. 2f) disruption of the preformed Aβ_40_ fibrils by treatment with a 5-fold excess concentration of SP1 and CBp, respectively.

We also performed TEM and Congo red staining experiments to examine the efficacy of SP1 disrupting the Aβ_40_ preformed fibrils. Equimolar or higher doses of SP1 disrupted the preformed fibrillar assembly of Aβ_40_, as confirmed by TEM (Fig. 2g). In comparison, CBp disrupted the Aβ_40_ fibrils when treated with a 2-fold or higher molar excess. Upon incubation with an equimolar concentration of SP1, a remarkable disappearance of the preformed Aβ_40_ aggregates was evident in the Congo red birefringence staining experiment (Fig. 2h). However, we observed a significant disappearance of birefringence only at a 5-fold molar excess of the control peptide (ESI Fig. 11b and c†). Notably, SP1 failed to demonstrate the efficacy of fibril disruption of Aβ_40_ at a 0.5-fold molar concentration. Collectively, these results strongly demonstrate that SP1 is more efficient than CBp in disaggregating fibrils of Aβ_40_.

### Inhibition of Aβ_42_ amyloid formation

The aggregation of Aβ_42_ causes significant neurotoxicity among all existing isoforms of Aβ.^35^ We, therefore, examined the inhibition efficacy of SP1 for Aβ_42_ aggregation. We performed similar biophysical experiments as described earlier for Aβ_40_. The ThT assay showed that the aggregation rate of Aβ_42_ was much faster than that of Aβ_40_ at a 50 μM concentration (Fig. 3a), and aggregation started immediately reaching a plateau within 20 h. ThT fluorescence intensity decreased in a dose-dependent manner by treating Aβ_42_ aggregates with SP1 (Fig. 3a and b). Around 84% inhibition of Aβ_42_ aggregation was observed when treated with a 5-fold molar excess of SP1 (Fig. 3b).

The aggregated Aβ_42_ in the absence of SP1 showed densely populated fibrillar structures under TEM (Fig. 3c), indicating the amyloid signature, as previously reported.^36^ With the assistance of SP1, the density of Aβ_42_ fibrils was reduced in a dose-dependent manner. Also, untreated Aβ_42_ aggregates exhibited green-gold birefringence upon staining with Congo red (Fig. 3d), and this signal was decreased in a dose-dependent manner by SP1. Collectively, these examinations indicate that the efficiency of SP1 for the inhibition of Aβ_42_ aggregates is comparable as observed with Aβ_40_.

### SP1 reduces Aβ aggregate-induced dye leakage from LUVs

Smaller Aβ oligomers or protofibrils are more toxic than mature fibrils in AD progression due to their ability to disrupt membranes *via* pore formation.^37,38^ Therefore, it is essential to examine whether SP1 can convert the toxic oligomeric species of Aβ into a non-toxic one. To evaluate this, we performed a membrane leakage assay on carboxyfluorescein-loaded large unilamellar vesicles (LUVs).^38^ The time required for Aβ_40_ oligomer and mature fibril formation is 12 h and 72 h, respectively (inferred from ThT assay, black curve, Fig. 2a), which directed us to set up the LUV leakage assay. Dye-loaded LUVs were incubated with the corresponding Aβ_40_ oligomers (12 h aged), mature fibrils (72 h), and freshly disaggregated Aβ_40_ fibrils in solution and SP1 or CBp (ESI Fig. 12b and c†). The fluorescence intensity of complete dye release from LUVs by Triton X-100 served as a positive control (100% leakage), and untreated dye-loaded LUVs assisted as a negative control. The Aβ_40_ oligomers (12 h aged) caused rapid dye leakage of ∼40% until 100 min (ESI Fig. 12b and c†), whereas mature Aβ_40_ fibrils caused ∼15% leakage and the untreated LUVs showed a minimal leakage of ∼9% (ESI Fig. 12b and c†) during the same period. These results establish that the Aβ_40_ oligomers trigger more dye leakage than the mature fibrils, and hence are likely to be more toxic.^38^

Membrane disruption by Aβ_40_ proceeds through a two-step mechanism.^39–41^ In the first step, Aβ_40_ monomers self-assemble to form soluble oligomers, which bind to the lipid membranes to form small ion-selective channel-like pores. During pore formation, the oligomers of Aβ_40_ further self-assemble and form larger aggregates that lead to the formation of mature fibrils which are released from the membrane. In the second step, the onset of Aβ_40_ aggregation and fibril formation causes membrane disruption through a detergent-like mechanism.^39–41^ Notably, freshly disaggregated Aβ_40_ fibrils by SP1 and CBp did not considerably damage the LUV membrane, evidenced by only ∼10% leakage from the LUVs, respectively, which is comparable to that from untreated LUVs. These results collectively affirm the potential ability of SP1 for disassembling preformed fibrils and other oligomers of Aβ_40_ to an innocuous species.

The LUV leakage assay was further employed to examine whether inhibition of Aβ_42_ fibril formation by SP1 leads to toxic soluble oligomers or not. Aβ_42_ (50 μM) was allowed to aggregate for 1 h, 5 h, 10 h, and 20 h in the absence or presence of 5-fold molar excess of SP1. Untreated Aβ_42_ samples, incubated for 1 h or 5 h, were considered oligomers, whereas 10 h or 20 h aged samples were considered mature fibrils, as inferred from the ThT assay results (black curve, Fig. 3a). In the absence of SP1, Aβ_42_ aggregates caused ∼60% (1 h aged), ∼55% (5 h aged), ∼23% (10 h aged) and ∼20% (20 h aged) leakage, respectively. This result indicates that Aβ_42_ oligomers are more toxic than the mature fibrils (10 h and 20 h aged) (Fig. 4a) and Aβ_42_ oligomers behave similarly to Aβ_40_. Then, SP1-treated Aβ_42_ samples were applied to LUVs and substantially less leakage was observed (∼40% (1 h aged), ∼15% (5 h aged), ∼13% (10 h aged) and ∼10% (20 h aged)) compared to the untreated Aβ_42_ samples (Fig. 4b), indicating that SP1 inhibited toxic oligomer or fibril formation.

### Monitoring early events by DLS and TEM

The inhibition of Aβ_42_ oligomer or fibril formation by SP1 at different time intervals was further examined using DLS (Fig. 4c and d) and TEM (Fig. 4e and f) to probe the early intermediates. The untreated Aβ_42_ samples, incubated for 1 h, 5 h, 10 h and 20 h, showed hydrodynamic diameters of ∼8 nm, ∼38 nm, ∼825 nm, and ∼940 nm, respectively (Fig. 4c), indicating the formation of oligomers (at 1 h or 5 h) and mature fibrils (at 10 h and 20 h), as observed in previous reports.^42,43^SP1 treated samples exhibited hydrodynamic diameters of ∼15 nm, ∼340 nm, ∼640 nm, and ∼730 nm, respectively (Fig. 4d). These results indicated that upon 5 h or more prolonged incubation of Aβ_42_ with SP1, it caused oligomer formation inhibition as smaller aggregates disappeared and were converted to larger, possibly amorphous aggregates. We further validated this phenomenon by TEM and observed that Aβ_42_ exhibited smaller aggregates at 1 h or 5 h. In contrast, dense fibrils appeared at 10 h or 20 h, suggesting oligomer formation at 1 h or 5 h, as observed in the DLS results. In the presence of SP1, we did not observe any fibrillar aggregates in all tested time intervals; instead, some amorphous aggregates were noted. These amorphous aggregates were non-toxic, as evident from the LUV experiments mentioned above and the cytotoxicity assay (*vide infra*). The formation of non-toxic amorphous species suggests that SP1 drives Aβ_42_ aggregation towards off-pathway aggregation in line with a previous report.^43^

### Prevention of Aβ_40_ induced cytotoxicity

Since the protofibrils of Aβ species induce cytotoxicity in neuronal cells,^44,45^ we investigated the inhibition potential of SP1 against Aβ_40_ induced neurotoxicity in human neuroblastoma SH-SY5Y cells as a cellular model system of AD.^29,30^ Initially, we explored the toxicity of SP1 and did not observe any discernible cytotoxicity even at the maximum concentrations (10 μM) used in the experiments (ESI Fig. 13a†). Further, the cells were incubated with 10 μM Aβ_40_ for 24 h in the absence or presence of graded concentrations of SP1 (0.5–10 μM). We observed a significant reduction in the cell population treated only with Aβ_40_ compared to the negative control and in the absence of SP1. However, the incubation of SP1 ameliorated the toxic effect considerably at 5 μM (∼82%), as determined by cell viability (ESI Fig. 13b†). Then, we explored the membrane damage induced by Aβ_40_ (ref. ^45^ and ^46^) using the lactate dehydrogenase (LDH) assay. Treatment of Aβ_40_ released a significant amount of cytosolic LDH into the culture medium of SH-SY5Y cells. Co-incubation of Aβ_40_ with SP1 at the respective concentrations (5 μM and 10 μM) revealed a substantial reduction in LDH leakage into the cell culture medium as compared to only Aβ_40_ treated cells (ESI Fig. 13c†). These two findings indicate that SP1 at a molar ratio of 1 : 2 (SP1 : Aβ_40_) is sufficient to demonstrate maximum inhibition of Aβ_40_ mediated cellular cytotoxicity. Interestingly, Aβ_40_ induced neuronal cell death was preserved for at least three days upon treatment with SP1 (5 μM) (ESI Fig. 13d and e†).

### SP1 ameliorates oxidative stress injury, apoptosis, and Ca^2+^ homeostasis

Condensed or fragmented nuclear bodies characterize the distinctive nature of apoptotic cells. To explore the anti-apoptotic and cytoprotective properties of SP1, we used Hoechst 33258 as a DNA staining dye. A significant number of apoptotic cells were observed under a fluorescence microscope when the cells were treated with Aβ_40_ (10 μM) for 24 h (Fig. 5a and b) compared to untreated cells. Upon co-incubation with SP1 (5 μM), the number of apoptotic cells holding damaged DNA was markedly reduced (Fig. 5b). These findings illustrate the potency of SP1 in regulating Aβ_40_ induced DNA damage in SH-SY5Y cells. The underlying mechanism of this neuronal apoptosis and oxidative damage has been reported to be significantly influenced by ROS generation, followed by triggering mitochondrial apoptotic events.^47,48^ In another experiment, we observed that the intensity of the ROS sensitive fluorescent marker in SH-SY5Y cells increased in the presence of Aβ_40_ compared to untreated cells, and the co-incubation of cells with Aβ_40_ and SP1 (ratio 1 : 2) significantly inhibited Aβ_40_ induced ROS production (Fig. 5c). Dyshomeostasis of Ca^2+^ is also responsible for the increased production of Aβ peptides, by which a degenerative feed-forward cycle is activated, resulting in accelerated apoptosis, synaptic dysfunction, and memory impairment.^49^ To examine the effect of Aβ_40_ with or without SP1 on Ca^2+^ homeostasis, we measured intracellular free Ca^2+^ using a fluorescent Ca^2+^ indicator, Fura-2AM. Our results demonstrated that Aβ_40_ (10 μM) significantly elevated intracellular Ca^2+^ levels in SH-SY5Y cells as compared to untreated cells. Then co-incubation with a molar ratio of 1 : 2 (SP1 : Aβ_40_) reduced Ca^2+^ levels to 98% compared to Aβ_40_ treated cells (Fig. 5d). The experiments showed that SP1 preserves Ca^2+^ dyshomeostasis induced by Aβ_40_*via* encumbering the oligomeric conversion of Aβ_40_.

### Effect of SP1 on Aβ_40_ induced apoptotic protein markers

Accumulation of Aβ triggers the generation of intracellular free radicals and leads to the activation of caspases *via* releasing cytochrome-c from mitochondria. Bcl-2 family proteins, pro-apoptotic Bax proteins, and caspases are well known to be involved in the mitochondrial apoptotic pathway.^50^

Western blot analyses of SH-SY5Y cells suggested that Aβ_40_ upregulates the level of Bax and causes a slight change in the Bcl-2 level, which is a significant increase in the ratio of Bax/Bcl-2 expression (∼3.2 fold) as compared to that in healthy cells. Interestingly, the expression of Bax protein was markedly downregulated by treatment with SP1 in SH-SY5Y cells for 24 h (Fig. 5e and f). Further, western blot analysis also revealed that the expression level of cleaved caspase-9 or caspase-3 significantly decreased after incubation with SP1 for 24 h (Fig. 5e, g and h). However, treatment with Aβ_40_ alone leads to activation of caspase-3 directed DNA breakage, nuclear chromatin condensation, and neurocellular apoptosis. These outcomes confirm the active suppression of Aβ_40_ mediated mitochondrial apoptosis and cell death by SP1 by inhibiting Aβ oligomer formation.

### Evaluation of acute and sub-chronic toxicity of SP1*in vivo*

We predicted the cytotoxicity of SP1 in Sprague-Dawley rats as per our previous report.^51^ A total of 24 rats were used in this study (*n* = 8/group) and divided into three groups: group1: control, no treatment; group 2: received 100 μg kg^−1^ of SP1 and group 3: received 500 μg kg^−1^ of SP1. The SP1 was administered into the tail vein for 42 days once in a day. We did not observe any cytoarchitectural changes in the liver and kidney tissue after the injections of two different doses in the groups (Fig. 6a). Interestingly, SP1 causes neither mortality nor abnormal behavioral patterns in rats. Besides, we did not observe any significant changes in the rats' body weight at two different doses (100 μg kg^−1^ and 500 μg kg^−1^) compared to their respective control groups on days 7, 21, and 42 by sub-chronic study (ESI Fig. 14†). Notably, we did not find any severe changes in the hematological and biochemical parameters in the group of rats treated with SP1 (100 μg kg^−1^) even after 42 days. Almost similar observations in rats treated with SP1 (500 μg kg^−1^) were revealed, except for monocyte and SGOT levels (ESI Table 3†). The tabulated biochemical profile (novel biomarkers of the liver and kidney) corroborates the safety charms of SP1 for further *in vivo* studies.

### SP1 ameliorates hippocampal neurodegeneration in rat brain

The overproduction of Aβ damages hippocampal neurons and causes cognitive impairments in AD. Previous data motivated us to explore the potential of SP1 in ameliorating hippocampal neurodegeneration. Cresyl violet staining was performed for identification of Nissl granules in neurons to reveal hippocampal neurodegeneration in this experiment. One-way ANOVA showed a significant contrast in the intensity of granules in the hippocampus between the groups [*F* (4, 25) = 102.4, *P* < 0.001].

Furthermore, Tukey's post hoc test suggested that the intra-hippocampal microinjection of toxic Aβ_40_ in hippocampal neurons showed a significant (*P* < 0.01) decrease in the intensity of Nissl granules (Fig. 6b) as compared to the control and sham groups, which indicates that neurons have degenerated. However, SP1 treatment at both the dosages (40 μM and 100 μM) in pre-Ab40 injected rats reduced the degeneration of hippocampal neurons significantly (*P* < 0.01), demonstrated by the intensity of Nissl granules (Fig. 6b). Hence, we established that SP1 treatment exhibits neuroprotective function against Aβ_40_ induced neurotoxicity.

### Investigation of the interaction between Aβ and SP1

High-resolution 2D Heteronuclear Multiple Quantum Coherence (HMQC) NMR experiments were performed with 80 μM Aβ40 with increasing concentrations of SP1 (titrated up to a molar ratio of 1 : 10). The Aβ_40_ backbone amide resonances resulted in concentration-dependent residue-specific chemical shift perturbations (CSPs) in the presence of SP1. At a molar ratio of 1 : 10, the molecular interaction resulted in notable CSPs, specifically for the central hydrophobic-K^16^LVFFA^21^ region (Fig. 7a). Similar observations were also made for the C-terminal region, particularly the I^31^IGL^34^ stretch and the hydrophobic V36, V39, and V40 residues (Fig. 7b). These observations clearly indicated the specific involvement of these hydrophobic-rich segments in the molecular association with SP1. Recent studies have highlighted the K^16^LVFFA^21^ segment to be essential for the Aβ_40_ fibrillation propensity.^52–55^ Extensive reports have provided evidence for the segment to be closely associated with the dock–lock mechanism underlying Aβ nucleation events. Thus, the SP1 mediated perturbation of this crucial domain suggests molecular interference in the dock–lock interactions of monomeric Aβ, explaining the altered fibrillation.^55,56^ Alternatively, the association of SP1 with the hydrophobic K^16^LVFFA^21^ and the C-terminal segments also stands to explain the reduced membrane damage and subsequent toxicity. These hydrophobic segments have been shown to internalize within the hydrophobic acyl region of the lipid membranes, disrupting the membrane integrity.^57^ Our recent studies have shown the crucial role played by the C-terminal residues in mediating cytotoxicity. Our mutation-based studies have suggested the role played by the GxxxG motifs from the C-terminal in aiding the helix–helix association and regulating the Ab fibrillation pathway.^58^ Thus, a direct molecular association of SP1 with these segments indicates the inaccessibility of these segments necessary for wild-type Aβ amyloidogenesis.

Interestingly, very similar observations were obtained for the residue-specific interaction studies between Aβ_42_ and SP1 (Fig. 7c). HMQC profiles showed significant CSPs, specifically involving the central K^16^LVFF^19^ segment and the C-terminal hydrophobic residues, including G29, G33, V36, I41, and A42 (Fig. 7d). The direct association of the terminal residues in Aβ_42_ is further reminiscent of the reduced cytotoxicity mediated upon SP1 interaction. Reports have found the increased C-terminal stability of Aβ_42_ to be entropically favorable for the cytotoxic fibrillation.^59^ Thus, high CSPs for the C-terminal residues in Aβ_42_ corroborate well with the functional implication of SP1 in modulating Aβ aggregation propensity.

Next, singular value decomposition (SVD) was used to obtain the residue-specific binding affinity of SP1 to Aβ_40_. The CSPs for Aβ_40_ with SP1 were adjusted for both Δ*δ*_N_ and Δ*δ*_H_ to extract the dissociation constant (*K*_D_) for each residue (see the NMR method and ESI Fig. 15–17† for details). Comparatively lower *K*_D_ values of ∼200 μM were obtained for the residues R5, L17, V24, K28, and G29 (ESI Table 4†) of Aβ_40_, indicating their functional unavailability in fibrillation. Once again, these data support the inhibition of the dock–lock mechanism of Aβ_40_ by SP1.

The lack of transferred NOE peaks (trNOEs) restricted us from determining the three-dimensional structure of SP1 bound to Aβ_40_ (data not shown). Although the designed paratope's (SP1) affinity is moderate, this is the first example of a synthetic paratope to prevent Ab aggregation. However, we are working further to improve the molecular association.

### Mechanistic investigation of Aβ_40_ engulfment by SP1

With the distinguished *in vitro* and *in vivo* activities, we explored the molecular alignment of SP1 and its mode of action in arresting Aβ_40_ peptides using the fluorescence resonance energy transfer (FRET) assay.^60–63^

To this end, different fluorophore-labeled synthetic paratopes, SP1A (donor), SP1B (acceptor), and SP1C (containing both), were prepared, along with the fluorescently labeled homologous Aβ fragments, LP1A (donor) and LP1B (acceptor) (ESI Table 1†).

**Fig. 8:**
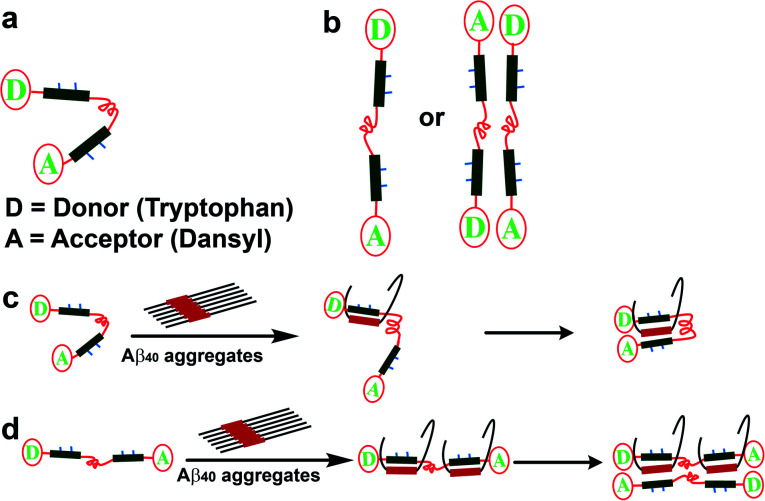
Predicted structural orientation of SP1 and plausible mode of interaction between Aβ_40_ and SP1: (a and b) plausible structural alignments of the SP1. (c and d) Predicted mode of engulfment of Aβ_40_ by SP1.

We considered the two most plausible structural alignments of SP1, hairpin-like or linear (Fig. 8a and b). Since we observed that SP1 is non-amyloidogenic (ESI Fig. 8†), self-aggregation with the hairpin-like conformation does not qualify. Now, if SP1 adopts a hairpin-like structure (Fig. 8a), it should exhibit intra-molecular FRET or intermolecular FRET through straight-chain alignment (Fig. 8b). Interestingly, SP1C showed a unique FRET event in contrast to the mixture of equimolar SP1A and SP1B, which did not show a significant change in emission. The time-resolved fluorescence study further confirmed a similar observation (ESI Table 5 and Fig. 18†). These combined pieces of information and the calculated Förster radius (*R*_0_)^60–63^ of the donor/acceptor system which was 27.9 Å (Section 1.2.j in the ESI†) further corroborate the U-shaped or hairpin-like structural alignment of SP1 (Fig. 1b and 8a). Also, we obtained direct evidence of interaction between SP1 and the homologous sequence of the Aβ_40_ peptide, resulting in substantial FRET events and positive implication of time-resolved fluorescence (ESI Table 6 and Fig. 19†). The aforementioned studies were conducted to comprehend the interaction of SP1A to LP1B and that of SP1B to LP1A by incubating equimolar concentrations. Data, revealed through all the present studies, allowed us to propose two plausible modes of interaction, which demonstrate inhibition of amyloid formation and disruption of preformed aggregates of Aβ_40_ by SP1 (Fig. 8c and d). The proposed models were further validated through the FRET, time-resolved fluorescence study, and Förster radius (*R*_0_) calculations.

Briefly, a significant FRET event was observed when SP1C was mixed in pre-captured Aβ_40_ with SP1 solution, and no FRET events resulted in the mixture (SP1A + SP1B) added to the pre-captured Aβ_40_ with SP1 solution (ESI Fig. 20 and Table 7†). We have noted earlier that the hairpin-shaped conformation of SP1 remains unaltered in the presence of Aβ_40_ aggregates. Therefore, these experiments together clarify that the U-shaped synthetic paratope prevents amyloid oligomer formation, most probably through the zipping action proposed in Fig. 8c.

To target a particular epitope of various amyloidogenic proteins including tau, Aβ, α-synuclein, and β_2_-microglobulin, and to antagonize their aggregation, Nowick and co-workers previously developed rationally designed 54-membered cyclic peptides, amyloid β-sheet mimics (ABSMs).^64–68^ The ABSM peptide was comprised of two strands, linked with two ornithine δ-linkages. One of the two strands was selected for recognition of the target amyloidogenic protein, whereas another strand contained an unnatural amino acid, “Hao” used as a β-sheet breaker unit to inhibit the aggregation of the target disease protein. Due to the cyclic structure, these peptides were less flexible yet effective in inhibiting the aggregation of various amyloidogenic proteins. In contrast, in the present study, we designed a hairpin-like flexible synthetic paratope comprising two epitope-binding peptides connected through a PEG linker. The synthetic paratope can bind to the target epitope of the Aβ peptide from both sides, and the presence of *N*-methylation on alternate amino acids does not allow Aβ monomers to self-assemble to form amyloids.

The structural design of ABSM containing a “Hao” unit helped the cyclic peptide prevent ABSMs from aggregating in solution to form a larger aggregated network of β-sheets; instead, it dimerized and further self-assembled into oligomers.^64^ In contrast, the synthetic paratope (SP1) did not self-assemble to form oligomers or larger aggregates due to the presence of *N*-methylation at alternate amino acids. In addition, the presence of PEG groups in the synthetic paratope, in contrast to the hydrophobic side chain of ornithine in ABSM, increases aqueous solubility, which is an essential factor from a therapeutic perspective. Moreover, due to the hairpin-like structure, the synthetic paratope exhibits more flexibility and possibly can show a better efficacy to inhibit the aggregation of Aβ or other amyloidogenic proteins than the existing peptide probe.

## Conclusion

In the present study, we have demonstrated the design, synthesis, and characterization of a synthetic paratope (SP1) that selectively binds with the epitope LVFFA, a vital amyloidogenic part of the Aβ peptide. A series of *in vitro* biophysical experiments, including NMR, support the inhibition of Aβ_40_ and Aβ_42_ aggregation by SP1 at an atomic resolution. SP1 was also equally efficient in disaggregating the preformed fibrillar assembly of the Aβ40 peptide into non-toxic species. We speculate that the synthetic paratope may further enable for designing an affinity tag for AD diagnosis, a reporter of the Aβ40 peptide, and a PROTAC type therapeutic against AD. The ability to ameliorate Aβ_40_ induced neurotoxicity, ROS generation, and apoptosis and maintain intracellular Ca^2+^ homeostasis of SP1 is remarkable for the further construction of suitable anti-apoptotic and anti-inflammatory peptide probes. The designed, non-toxic synthetic paratope may gain considerable attention for ameliorating Aβ-induced hippocampal neurodegeneration, corroborated by preliminary *in vivo* studies in a rat model of AD. In-depth investigations with animals may be carried out after improving its binding affinity to the nanomolar level, which is at the micromolar range at present. Further improvement of solubility and enzymatic stability is also required.

We believe that the dissected zipping-mechanism for capturing the Aβ_40_ peptide by a synthetic paratope will significantly facilitate the design of a great variety of paratopes. Such a smartly designed molecular construct may also find applications in diverse directions spanning chemical biology, diagnostics, and therapeutics. Our findings suggest that due to the structural flexibility and moderate to weak affinity towards the target epitope, the synthetic paratope might lead to potential hit discoveries against Alzheimer's disease, extendable further to other amyloidoses.

## Conflicts of interest

The authors declare no conflict of interest.

## Supplementary Material

SC-012-D0SC04379F-s001
