# Supplementary material for: An explicitly designed paratope of amyloid-β prevents neuronal apoptosis in vitro and hippocampal damage in rat brain
Source: Chem Sci. 2020 Dec 22;12(8):2853–62. doi: 10.1039/d0sc04379f (PMC8179358; doi:10.1039/d0sc04379f)
Supplement: SC-012-D0SC04379F-s001 [file SC-012-D0SC04379F-s001.pdf]

Electronic Supplementary Material (ESI) for Chemical Science.
This journal is © The Royal Society of Chemistry 2020

Supplementary Information

An explicitly designed paratope of Amyloid-β prevents neuronal apoptosis in vitro and hippocampal damage in rat brain

Ashim Paul[a], Sourav Kumar[b], Sujan Kalita[a], Sourav Kalita[a], Dibakar Sarkar[c], Anirban Bhunia[c], Anupam Bandyopadhyay[d], Amal Chandra Mondal[b,e*] and Bhubaneswar Mandal[a*]

[a]Laboratory of Peptide and Amyloid Research, Department of Chemistry, Indian Institute of Technology Guwahati (IITG), North Guwahati, Assam-781039, India

[b]Neuroscience Research Unit, Department of Physiology, Raja Peary Mohan College, Uttarpara, Hooghly, West Bengal-712258, India.

[c]Biomolecular NMR and Drug Design Laboratory, Department of Biophysics, Bose Institute, P-1/12 CIT Scheme VII (M), Kolkata 700054, India.

[d]Biomimetic Peptide Engineering Laboratory, Department of Chemistry, Indian Institute of Technology, Ropar, Punjab-140001, India.

[e]Laboratory of Cellular & Molecular Neurobiology, School of Life Sciences, Jawaharlal Nehru University, New Delhi-110 067, India.

Correspondence to: Dr. Bhubaneswar Mandal, E-mail: bmandal@iitg.ac.in, Dr. Amal Chandra Mondal, E-mail: acmondal@mail.jnu.ac.in,

Table of Contents

Contents **Page No.**

1. Materials and methods **4-23**

1.1. Reagents, peptide synthesis and their characterization 4

1.2. Methods for *in vitro* biophysical experiments 6

a. Sample preparation 6

b. Thioflavin T Fluorescence Assay 7

c. Transmission Electron Microscopy (TEM) 7

d. Congo-Red Stained Birefringence 7

e. Time resolved photoluminescence (TRPL) 8

f. Dynamic light scattering (DLS) 8

g. Carboxyfluorescein dye loaded Large Unilamellar Vesicles (LUVs) preparation 8

h. Carboxyfluorescein dye loaded Large Unilamellar Vesicles (LUVs) Leakage Study 9

i. NMR experiments and SVD analysis 9

j. Förster resonance energy transfer (FRET) study 11

k. FRET study for the evidence of proposed mechanism 15

1.3. Methods for in vitro cell-based experiments 16

a. Cell Culture 16

b. MTT Assay 17

c. LDH Release Assay 18

d. Measurement of intracellular ROS 18

e. Measurement of intracellular calcium (Ca^{2+}) 19

f. Western Blot Analysis 19

1.4. Methods for *in vivo* animal-based experiments 20

a. Animals 20

b. Design of experiments 21

c. Preparation of SP1 and Administration of SP1 into the tail vein 21

d. Analysis of blood for hematological and biochemical parameters in respect to SP1 toxicity 21

e. Histopathological experiments for sub-chronic toxicity 22

f. Preparation of stock toxic Aβ_{40} and SP1 22

g. Intra-hippocampal microinjection of toxic Aβ_{40} and SP1 by stereotaxic surgery 22

h. Stereotaxic Surgery 23

i. Cresyl violet staining of hippocampal neurons to assess neuroprotective effects of SP1 24

2.List of all the peptides, general synthetic scheme and their characterization data 24-27

3. Additional spectra and figures 28-42

4. References 43

1. Materials and Methods

1.1. Reagents

MBHA-Rink amide resin (Loading 1.1 mmol/g), BOP [(Benzotriazole-1-yloxy) tris (dimethylamino) phosphoniumhexafluorophosphate], Diisopropylethylamine (DIPEA), Dansyl Chloride, Fmoc-L-2,3-diaminopropionic acid, 5(6)-Carboxyfluorescein, ThioflavinT, Congo red, Retionic acid (RA), protease inhibitor cocktail, 3-(4,5-dimethylthiazol-2-yl)-2,5-diphenyltetrazolium bromide (MTT) and 2′,7′-dichlorodihydrofluoresceindiacetate (H2DCF-DA) were purchased from Sigma-Aldrich. Fetal bovine serum (FBS), DMEM/F_{12} media, antibiotic, and trypsin-EDTA for cell culture were from Gibco/BRL life-technologies (Grand Island, NY, USA). Antibodies against caspase-3, Bcl-2, Bax were from Cell Signaling Technology (MA, USA). Dimethylformamide (DMF), dichloromethane (DCM), Diethylether, and acetonitrile (HPLC grade) were obtained from Merck (India). 1,2-Dimyristoyl-sn-glycero-3-phosphocholine (DMPC), Ganglioside GM1 were purchased from Avanti Polar Lipid, Inc. Cholesterol (99%), acetic anhydride, *N*-methyl imidazole, Trifluoroacetic acid (TFA) were purchased from SRL (India). Milli-Q water at 18.2 Ω was used. All solvents were extra pure and analytical grade. Fmoc amino acids, human $A\beta_{40}$ and $A\beta_{42}$ were purchased from GL Biochem (Shanghai).

Peptide Synthesis

The Synthetic paratope was synthesized on 300 mg of Rink amide MBHA resin (loading 1.1 mmol/g) using standard Fmoc/^{t}Bu solid-phase peptide synthesis method. We first synthesized one zipper strand of the paratope, fragment **Peptide 1** (HO-Adi-Gly-(*N*-Me)Leu-Val-(*N*-Me)Phe-Phe-Ala-NH_2, Adi = Adipic acid) separately and attached to another strand, fragment **Peptide 2,** a resin-bonded peptide (H-PEG_3-Suc-Gly-Leu-(*N*-Me)Val-Phe-(*N*-Me)Phe-Ala-Resin (Suc = Succinic acid)) using PyBOP (3 equivalents) and DIPEA (7 equivalents) to get the desired synthetic paratope **SP1 (Supplementary Scheme 1)**, in which the two zipper strands were connected through PEG. Each coupling was performed with two equivalents

of Fmoc amino acid, 2.5 equivalents of BOP, and five equivalents of DIPEA. After each coupling, the resin was washed with DMF and DCM for 5 min each (5 x 1 min), and Kaiser test was performed. Fmoc group was removed using 20% piperidine in DMF mixture for 21 min (7 min x 3). The final product **SP1** was cleaved from the resin using a mixture of TFA/DCM/H_2O (9/0.5/0.5) 3 mL for 3h. The crude peptide was precipitated using cold diethyl ether. Due to hydrophobicity in the peptide precipitate was very less. Therefore, the excess ether was evaporated and obtained a crude product. The other peptides were also prepared similarly.

Purification

Crude peptides were dissolved in CH_3CN/H_2O and purified by RP-HPLC (Waters 600E) using a C18 Bondapak column at a flow rate of 5 mL/min. The binary solvent system was used, solvent A (0.1% TFA in H_2O) and solvent B (0.1% TFA in CH_3CN). A Waters 2489 UV detector was used with an option of dual detection at 214 and 254 nm. A linear gradient was used from 5 to 20% CH_3CN till 2 min and 20 to 100% CH_3CN till 18 min in a total run time of 20 min) and lyophilized to obtain pure product.

Mass Spectrometry

The mass of the synthesized peptides was analyzed on Micromass Q-TOF equippedwith Masslynx software in ESI positive mode.

Characterization of Non-amyloidogenicity of the SP1

To examine the inherent amyloidogenicity of **SP1**, we incubated a 100 µM solutions of **SP1** and **CBp** in PBS (50mM, pH 7.4) at 37 °C for seven days. **SP1** showed a β-sheet rich conformation in CD (Supplementary Fig. 8a) and FTIR (Supplementary Fig.8b) spectroscopic analyses. However, there is no characteristic evidence of fibrillar aggregates under TEM (Supplementary Fig. 8c); under cross-polarized light after staining with Congo red does not led green-gold birefringence (Supplementary Fig. 8d). These results collectively indicate that **SP1** forms a β-sheet structure, but non-amyloidogenic in nature. CD, FTIR, TEM, and birefringence analyses with the control peptide (**CBp**) revealed asimilar outcome (Supplementary Fig. 9).

Circular Dichroism (CD)

400μL of the sample was taken in a cuvette of 1mm path length. Three measurements were accumulated. Spectra were recorded from 190nm to 260nm on a JASCO J-1500 instrument. Observed ellipticity (mDeg) was converted to mean residue molar ellipticity using the equation:θ (deg. cm^2 .$dmol^{-1}$) = Ellipticity (mdeg). 10^6 / Pathlength (mm). [Peptide] (μM). N (no. of peptide bond).

Fourier Transformation Infrared (FT-IR)

An aliquot of 20μL was taken at the required time, mixed with KBr, and a pellet was prepared. The background scan was subtracted from the sample scans, and text files were plotted using OriginPro 8 software.

1.2. Methods for *in vitro* experiments

a. Sample preparation:

1.5 mg of commercially available $A\beta_{40}$ peptide was taken and disaggregated by TFA (50μL) and evaporated under nitrogen gas. Later, to remove excess TFA, HFIP (50μL) was added to that and dried under nitrogen gas. The process was repeated thrice to obtain disaggregated film of $A\beta_{40}$peptide. The disaggregated material dissolved in 1.8 ml of PBS (50 mM, pH 7.4) by sonication and vortex to obtain a transparent solution and the whole solution was divided into 9 equal portions followed by addition of 600 μL of PBS to each portion to obtain a final $A\beta_{1-40}$ concentration of 50 μM. For the inhibition study, 0.5-, 1-, 2- and 5- fold molar excess of the inhibitors (synthetic paratope and control peptide) were mixed with each portion (800 μL) of $A\beta_{1-40}$ solution in the presence of 3% DMSO and kept for incubation over a water bath for seven days at 37 °C. To obtain precise results, two more replicate solutions of each portion were prepared (total number of samples for this experiment was 27), and fibrillization was monitored by various biophysical tools. The sample for $A\beta_{42}$ peptide was prepared in a similar manner as described for $A\beta_{40}$ peptide.

b. Thioflavin T Fluorescence Assay

A stock solution of Thioflavin T (ThT) of concentration 50μM in PBS (50mM, pH 7.4) was prepared and stored at 4 °C with the proper protection to prevent degradation from light. For inhibition studies,50μM of $A\beta_{40}$peptide alone and with different ratios (1:0.5, 1:1, 1:2, and 1:5) of synthetic paratope in PBS (pH 7.4) was incubated at 37 °C on a water bath. For fluorescence studies, 40μLof peptide sample was mixed with 200μL of ThT solution (50μM), and the total volume was made up to 400μL using PBS. Fluorescence emission was measured at 485nm and excitation at 440nm, using a slit of 5nm on a Fluoromax-4, Horiba instrument. The ThT kinetics with $A\beta_{42}$ peptides was performed in 96-well black plate (100 μL in each well) and the final concentration was 50 μM. The data were collected (in triplicates) using Infinite M200 microplate reader (Tecan, Switzerland), with measurements taken at 15 min intervals for 20 h. Excitation and emission wavelengths of ThT were 440 and 485 nm, respectively.

c. Transmission Electron Microscopy (TEM)

10μL aliquot from the stock peptide solution after incubation of 10 days (7 days for inhibition study) was added over the carbon-coated copper grid. Then 2% uranyl acetate solution (10μL) was added onto it and allowed to float for 1 min. The excess solution was removed using a blotting paper. The sample was dried at room temperature and kept in desiccators before taking TEM analysis on JEOL (Model: JEM 2100) instrument at 200kV.

d. Congo-Red Stained Birefringence

A 20μL aliquot from the stock was placed over a glass slide followed by 20μL of the saturated Congo red solution. The excess solution was removed using a blotting paper, dried at room temperature, and analyzed under a Leica ICC50 HD polarizable microscope.

e. Time-resolved photoluminescence (TRPL)

We used TRPL study to measure the lifetime of a fluorophore. The peptide samples (**SP1A**, **SP1B,** and **SP1C**)) were dissolved in PBS (in the presence of 3% DMSO) to make a solution of concentration ~20 μM. Then, 600 μL of peptide sample was placed in a 1 mL fluorescence cuvette and measure the excited state lifetime on Eddinburg FSP920 Instrument.

f. Dynamic light scattering (DLS) measurements

DLS[1,2] measurements were performed with the different $A\beta_{42}$peptide samples (oligomers and mature fibrils) on a Malvern nano zetasizer (Malvern, UK) using a laser source with λ=633 nm and a detector at a scattering angle of θ = 173 degrees. $A\beta_{42}$ peptide (50 μM) was incubated in a similar manner as described for the ThT assay, in the absence of ThT dye, for 1h, 5h, 10h and 20h min to generate oligomers and mature fibrils, respectively and the hydrodynamic diameter of them was immediately examined by DLS. Prior to the analysis, samples were filtered through 0.45 μm PVDF membrane. The samples were placed in a disposable cuvette and held at the corresponding temperature during the analysis. For each sample, the analyses were recorded three times with 11 sub-runs using the multimodal mode. The Z-average diameter was calculated from the correlation function using Malvern technology software.

g. Carboxyfluorescein dye loaded Large Unilamellar Vesicles (LUVs) preparation

LUVs were prepared using a mixture of DMPC, Cholesterol, and GM1 (68:30:2, molar). Lipids were dissolved in chloroform and methanol (2:1, 2 mM) in a glass vessel, and solvents were evaporated using nitrogen gas to make lipid films.[3] The lipid films were hydrated with 500 μL of carboxyfluorescein (CF) solution (200 μM) in 50mM HEPES buffer of pH 7.4. Then, the solution was vortexed vigorously for 30 min for emulsification. Then the glass vial was dipped into the liquid nitrogen for instant cooling, and after 5 min the frozen solution was dipped into a water bath at 50-60 °C for freeze-thawing.[4]This step was repeated five times. The excess dye was removed by ultracentrifugation at 20000 rpm, the supernatant

was discarded, and the lipid pellet was re-hydrated with 50 mM HEPES buffer. This step was repeated twice, and the final lipid pellet was collected followed by the addition of 500 µL of HEPES buffer and vortexed to obtain a homogenous suspension, which was filtered through 0.45 µm polycarbonate membrane to get the 2 mM stock of dye loaded LUVs. The formation of LUVs was characterized by TEM.

h. Carboxyfluorescein dye loaded Large Unilamellar Vesicles (LUVs) Leakage Study

For leakage study, peptide and lipids were taken in 1:20 molar ratios, and the dye release was monitored by fluorescence emission. Emission was measured at 516 nm, and excitation at 485 nm, 3 nm of bandwidth on a Fluoromax-4, Horiba instrument. The complete dye release from the vesicle was obtained using Triton X-100, and the final fluorescence was measured. The % leakage was calculated as,

$$\%\,Leakage = \frac{(Observed\ fluorescence - initial\ fluorescence)}{(Total\ fluorescence - initial\ fluorescence)} \times 100\ \% \quad \ldots\ldots\ldots\ldots\ldots\ldots\ldots\ldots (4)$$

From the instrument, the text file was taken, and the graph was plotted using Origin Pro 8 software. Each data point was the average of 3 independent scans.

Data Availability: The data sets generated and analyzed during the current study are available from the corresponding author upon reasonable request.

i. NMR experiments

The structural insight into the atomic level interaction between Aβ_{40} and **SP1** was obtained from 2D band-Selective Optimized-Flip-Angle Short-Transient (SOFAST) Heteronuclear Multiple Quantum Coherence (HMQC) NMR experiment.2D band-Selective Optimized-Flip-Angle Short-Transient (SOFAST) heteronuclear multiple quantum coherence (HMQC) spectra were acquired on a Bruker Avance III 500 MHz spectrometer equipped with a 5mm SMART probe with 48 scans and eight dummy scans at 283K

for each titration of **SP1** to either uniformly ^{15}N labeled Aβ40 or Aβ42. The spectral width was fixed to 12 and 28 ppm, with the offset at 4.7 ppm and 118 ppm, respectively for proton and nitrogen dimensions. Topspin 3.1 (Bruker Biospin software suite) and SPARKY 3.113 software (UCSF) were used for processing and analysis, respectively, of 2D spectra using previously available assignments for Aβ40 as a guide.

The chemical shift perturbations (CSPs) of selective residues were globally fitted using single-site binding and non-specific binding models, though the fittings did not give reliable dissociation constant (K_D) values. To get noise-free reliable fits and to estimate the number of independent states, singular value decomposition (SVD) was applied on the adjusted CSPs as well as for both $\Delta\delta_N$ and $\Delta\delta_H$. The adjusted chemical shift δ_{av}, calculated as $\delta_{av} = [\delta_H^2 + (\delta_N/5)^2]^{1/2}$ (Supplementary Fig. 15-17). For the titration of ^{15}N-$A\beta_{40}$with unlabeled **SP1,** the SVD analysis showed that the first two components have significantly higher singular values, and their corresponding v_i vectors have smooth shapes (Supplementary Fig. 15-17). Thus, the SVD analysis of the raw chemical shift perturbation dataset indicates two non-noise components. Then the noise-filtered dataset was reconstructed using two non-noise components, and the curves were fitted using single-site binding and non-specific binding models, from which the equilibrium dissociation constant was extracted with the more reliable fit (Supplementary Fig. 17).

SVD analysis: In the SVD analysis, we used the chemical shift difference referenced to the free form, $\Delta\delta_{av} = \delta_{obs} - \delta_F$, as a one-dimensional column vector. The two-dimensional dataset A was obtained by placing such column vectors for all n titrations subsequently. The SVD algorithm expresses the data matrix A in the following form:

$$A = USV^T$$

Where U is an M × N matrix, S is an N × N diagonal matrix, and V^T is also an N × N matrix.The columns of U are called the left singular vectors, u_i.The rows of V^T contain the elements of the right singular

vectors, v_i. The elements of S are only nonzero on the diagonal and are called the singular values. By convention, the ordering of the singular vectors is determined by high-to-low sorting of singular values, with the highest singular value in the upper left index of the S matrix.

In the ideal case without noise, the rank of the matrix A corresponds to the numberof mathematically independent states. However, for real data with noise,the number of non-noise components must be estimated by takinginto account (1) the singular values, (2) the shape of the v_i vectors, and (3) the RMSD of the SVD reconstruction. Once the number of non-noise components is determined, it ispossible to reconstruct the data set using only the non-noise components and discarding the noise components.

j. Förster resonance energy transfer (FRET) study

To performed FRET study, all the fluorophore containing peptides were dissolved in the absence and presence of $A\beta_{40}$ in PBS pH 7.4 (in the presence of 3% DMSO), and various photo-physical studies were carried out. The Förster radius (R_0) was calculated using equation 1,[5]

$$R_0 = 0.2108 \left[\kappa^2 \Phi_D n^{-4} \int_0^{\infty} F_D(\lambda) \varepsilon_A(\lambda) \lambda^4 d\lambda \right]^{1/6} \text{..........................(1)}$$

Where κ^2 (= 2/3) is the orientation factor, n is the refractive index of the medium (= 1.33), Φ_D is the quantum yield of the donor (**SP1A**) in the absence of acceptor and $\int_0^{\infty} F_D(\lambda) \varepsilon_A(\lambda) \lambda^4 d\lambda$ is the overlap integral of the fluorescence emission spectrum of the donor (**SP1A**) and the absorption spectrum of the acceptor (**SP1B**). R_0 is the critical distance when the energy transfer efficiency is 50%.

The fluorescence quantum yields (Φ_f)[6] were determined using equation 2, where quinine sulfate was used as a reference with the known Φ_f (0.546) in 0.1 molar solutions in sulphuric acid.

$$\Phi_S = \Phi_R \frac{Fl_S^{Area} \cdot Abs_R \cdot n_S^2}{Fl_R^{Area} \cdot Abs_S \cdot n_R^2} \text{............................(2)}$$

Where, Φ_R is the quantum yield of standard reference, Fl^{Area}_S (sample) and Fl^{Area}_R (reference) are the integrated emission peak areas, Abs_S. (sample) and Abs_R (reference) are the absorbance at the excitation wavelength, and n_S(sample) and n_R (reference) are the refractive indices of the solutions.

Again, the efficiency of energy transfer (E)[6] was calculated using equation 3,

$$E = \frac{R_0^6}{R_0^6 + r^6} = 1 - \frac{F}{F_0} \text{..........................}(3)$$

Where F and F_0 are the fluorescence intensity of donor in the presence and absence of acceptor, r is the distance between the donor and the acceptor, and R_0 is the Förster radius.

To gain insight into the mode of action of the synthetic paratope to arrest $A\beta_{40}$ peptide, fluorescence resonance energy transfer (FRET)[5-9] analysis was performed. We prepared three different fluorophore-labeled derivatives of the synthetic paratope, **SP1A** (contains tryptophan as FRET donor), **SP1B** (contains dansyl as FRET acceptor) and **SP1C** (contains both tryptophan and dansyl as FRET partners) and two short fluorescently labeled homologous Aβ fragments, **LP1A** (containing tryptophan as FRET donor) and **LP1B** (containing dansyl as FRET acceptor). Sequences of all these peptides are tabulated in Supplementary Table 1.

For FRET analysis, we prepared ~20 μM solutions of all the fluorophore-conjugated peptides (**SP1A**, **SP1B**, **SP1C, LP1A**, and **LP1B**) in PBS pH 7.4 (50 mM, in the presence of 3% DMSO). The UV-visible and fluorescence spectra of the individual donor (**SP1A**) and acceptor (**SP1B**) revealed that the fluorescence spectrum of dansyl in acceptor, **SP1B**, overlapped significantly with the absorption spectrum of tryptophan in the donor, **SP1A**(Supplementary Figure 18a). Importantly, **SP1C** contained both the donor (tryptophan) and acceptor (dansyl), which can be selectively excited at 290 nm (the absorbance maximum of the donor, **SP1A**) where there was very low absorbance of the acceptor **SP1B** (Supplementary Figure 18b). Therefore, these two fluorophores (tryptophan and dansyl) should form a

FRET pair in the designed peptide **SP1C** where the conceptual donor (tryptophan) and the acceptor (dansyl) acted as a FRET donor and acceptor, respectively. Critical criteria to exhibit significant FRET includes the energy transfer from the donor to the acceptor and the decrease of the fluorescence of the donor (both intensity and lifetime), whereas the fluorescence of the acceptor is expected to increase.

We observed that the fluorescence intensity of the donor (tryptophan in **SP1C**, λ_{max}^{Em} = 355 nm) in the presence of acceptor decreased almost 10 to 11 times from that of the emission of donor alone (**SP1A**, λ_{max}^{Em}= 355 nm) when it was excited at the maximum absorbance of the donor (λ_{max}^{Abs} = 290 nm). Simultaneously, the fluorescence intensity of the acceptor (in the presence of donor) in the **SP1C** increased almost 1.5 times than that of the fluorescence of the acceptor (**SP1B**, λ_{max}^{Em} = 495 nm). This clear change in fluorescence intensity provided visual evidence of the FRET process from the donor (tryptophan) to acceptor (dansyl). Further, to verify whether the FRET observed from the intra-molecular or inter-molecular, we mixed **SP1A** and **SP1B** (donor and acceptor both were present in the same solution but in different molecules) at the same concentration (~20 μM) and FRET analysis was performed. If the observation of FRET appeared through inter-molecular interaction, i.e., anti-parallel straight-chain interaction, we would expect FRET from the mixture (**SP1A** + **SP1B**). But, we did not observe any such occurrence, rather we noticed the fluorescence intensity of the donor in the mixture (tryptophan in **SP1A** + **SP1B**) was almost unchanged in comparison to the emission of donor alone (**SP1A**, λ_{max}^{Em} = 355 nm) when it was excited at the maximum absorbance of the donor (λ_{max}^{Abs} = 290 nm). Also, the fluorescence intensity of the acceptor in the mixture (dansyl, in **SP1A** + **SP1B**) did not increase in comparison to the fluorescence of the acceptor (dansyl in **SP1B**, λ_{max}^{Em} = 495 nm). This visual observation indicated no occurrence of FRET (Supplementary Figure 18c).

From the time-resolved fluorescence study, we observed that the donor lifetime (tryptophan in **SP1A**, λ_{ex} = 290 nm, λ_{em} = 355 nm) was decreased from 2.9 ns to 1.8 ns in **SP1C**, indicating the occurrence of the

FRET process. Moreover, the lifetime of the acceptor (dansyl in peptide **SP1B**, λ_{ex} = 290 nm, λ_{em} = 495 nm) was increased in **SP1C** from 6 ns to 15 ns (Supplementary Table 5 and Supplementary Fig. 18d) which was also an indication of FRET. However, in the mixture (**SP1A** + **SP1B**), the lifetime of the donor (2.9 ns) did not change from that of the donor in peptide **SP1A** (2.9 ns). Similarly, the lifetime of the acceptor in the mixture (**SP1A** + **SP1B**) was found 5.5 ns in comparison to the lifetime of the acceptor (**SP1B**) alone, which was 6 ns. The lifetime data also indicated no occurrence of intermolecular FRET in the mixture (**SP1A** + **SP1B**).

Absence of notable FRET through intermolecular interaction of the synthetic paratope, **SP1** did not satisfy the straight-chain orientation, whereas, observation of the intramolecular FRET indicated the U-shaped or hairpin-like structural orientation of **SP1**.

The Förster radius (R_0) of the tryptophan/dansyl system in **SP1C** was calculated using Equation 1 and found to be 27.9 Å (the overlap integral value = 4.206 x 10^{14} and the quantum yield of the donor = 0.06). Also, the efficiency of energy transfer (E) was calculated using Equation 3 and found to be 91.7 %. The donor-acceptor distance (r) in the synthetic paratope, **SP1C** was found to be 18.7 Å, which is far less than their theoretical distance when the peptide is in fully stretched conformation, which confirms its U shaped structure.

To get direct evidence of interaction between the **SP1** and $A\beta_{40}$ peptide, we mixed **SP1A** and **LP1B** as well as **SP1B** and **LP1A** (donor and acceptor both were present in the same solution but in different molecules) at the same concentration (~20 μM) in parallel. From the FRET analysis of the pair of **SP1A** and **LP1B**, we noticed that the fluorescence intensity of the donor (tryptophan) in the mixture (**SP1A** + **LP1B**,λ_{max}^{Em} = 355 nm, Supplementary Figure 19c) decreased considerably in comparison to the emission of donor alone (tryptophan in **SP1A**, λ_{max}^{Em} = 355 nm) when it was excited at the maximum absorbance of the donor (λ_{max}^{Abs} = 290 nm). Simultaneously, the fluorescence intensity of the acceptor (dansyl) in the

mixture (**SP1A** + **LP1B**, λ_{max}^{Em} = 495 nm) increased considerably in comparison to the fluorescence of the acceptor alone (dansyl in **LP1B**, λ_{max}^{Em} = 495 nm).

Similarly, for the other pair, we noticed the fluorescence intensity (Supplementary Figure 19d) of the donor (tryptophan) in the mixture (**SP1B** + **LP1A,**λ_{max}^{Em} = 355 nm) decreased considerably in comparison to the emission of donor alone (tryptophan in **LP1A**, λ_{max}^{Em} = 355 nm) when it was excited at the maximum absorbance of the donor (λ_{max}^{Abs} = 290 nm). Also, the fluorescence intensity of the acceptor (dansyl) in the mixture (**SP1B** + **LP1A**,λ_{max}^{Em} = 495 nm) increased considerably in comparison to the fluorescence of the acceptor alone (dansyl in **SP1B**, λ_{max}^{Em} = 495 nm). These results collectively indicated **SP1** indeed bound to the homologous sequence of $A\beta_{40}$.

Moreover, for the pair (**SP1A** and **LP1B**), we observed that the donor lifetime (tryptophan in **SP1A**, λ_{ex} = 290 nm, λ_{em} = 355 nm) was decreased from 2.07 ns to 1.47 ns in (**SP1A** + **LP1B**), indicating the occurrence of the FRET process. Also, the lifetime of the acceptor (dansyl in **LP1B**, λ_{ex} = 290 nm, λ_{em} = 495 nm) was increased from 4.06 ns to 4.37 ns in (**SP1A** + **LP1B**), which was also an indication of FRET. Similarly, for the pair (**SP1B** and **LP1A**), we observed that the donor lifetime (tryptophan in **LP1A**, λ_{ex} = 290 nm, λ_{em} = 355 nm) was decreased from 1.90 ns to 0.74 ns in (**SP1B** + **LP1A**), indicating the occurrence of the FRET process. Moreover, the lifetime of the acceptor (dansyl in **SP1B**, λ_{ex} = 290 nm, λ_{em} = 495 nm) was increased from 5.21 ns to 7.23 ns in (**SP1B** + **LP1A**), which was also an indication of FRET (Supplementary Table 6 and Supplementary Fig. 19a,b).

k. FRET study for the evidence of the proposed mechanism

To confirm the proposed mechanism, we added **SP1** (2-fold molar excess) to $A\beta_{40}$, co-incubated them for 150 h at 37 °C and performed further FRET studies. Similar to the previous results, we observed an intra-

molecular FRET from the synthetic paratope (**SP1C,** donor, and acceptor were present in the same molecule but at the opposite end) in the presence of Aβ_{40}. But, we did not observe any intermolecular FRET from the mixture (**SP1A** + **SP1B**, donor and acceptor were present in the same solution but in different molecules).

The Förster radius (R_0) of the tryptophan/dansyl system in synthetic paratope **SP1C** in the presence of Aβ_{40} peptide was calculated and found to be 28.2 Å (the overlap integral value = 3.629 x 10^{14} and the quantum yield of the donor = 0.075). The efficiency of energy transfer (E) was found to be 87 %. The donor-acceptor distance (r) in **SP1C** in the presence of Aβ_{40} aggregates was found to be 20.6 Å. Again, from the time-resolved fluorescence study, we observed that the donor lifetime (tryptophan in **SP1A**, λ_{ex} = 290 nm, λ_{em} = 355 nm) decreased from 2.6 ns to 1.6 ns in **SP1C**, indicating the occurrence of the FRET process. Moreover, the lifetime of the acceptor (dansyl in **SP1B**, λ_{ex} = 290 nm, λ_{em} = 495 nm) increased in **SP1C** from 13.4 ns to 14.9 ns (Supplementary Table 7 and Supplementary Fig. 20) which was also an indication of the occurrence of FRET. But in the mixture (**SP1A** + **SP1B**), the lifetime of the donor remained same (2.6 ns) in comparison to the donor alone in peptide **SP1A** (2.6 ns) and also the lifetime of the acceptor in the mixture (**SP1A** + **SP1B**) found to be similar, 13.1 ns, in comparison to the lifetime of the acceptor alone, 13.4 ns. The lifetime data also indicated no occurrence of FRET in the mixture (**SP1A** + **SP1B**).

We already observed that the **SP1** exists in hairpin-shaped structural orientation, which remained similar in the presence of Aβ_{40} aggregates also. Since **SP1** effectively inhibited the aggregation of Aβ_{40} and disrupted the existing aggregates, it can be inferred that the interaction between the **SP1** and the Aβ_{40} aggregates occurred. Moreover, we observed the occurrence of intramolecular FRET in the **SP1** in the presence of Aβ_{40} fragment as well as Aβ_{40} aggregates. These results altogether indicated that the interaction between the synthetic paratope and the Aβ_{40} occurred through zipping action.

1.3. Methods for *in vitro* cell-based experiments

a. Cell Culture

The human neuroblastoma cell line SH-SY5Y was obtained from the National Centre for Cell Science (NCCS, Pune, India). The cells were grown in DMEM, and Ham's F12 nutrient mixture (DMEM/F12; 1:1) supplemented with 10% fetal bovine serum (FBS; Gibco, USA), 50 U/ml penicillin, 100 µg/ml streptomycin and incubated in a humidified atmosphere of 5% CO_2 at 37°C. All experiments were carried out 24–36 h after seeded, and cells were reached with 90% confluency. Each experiment was performed in triplicate manner.

b. MTT assay

Methylthiazol tetrazolium (MTT) reduction assay was used to measure the viability of cells.[10] The SH-SY5Y cells were seeded at a density of 1.5×10^4 cells/well in a 96 well plate. To assess self-toxicity, cells were pre-incubated with different concentrations of SP1 (0.5, 1, 2.5, 5, and 10µM) for 24h. After that, 10µM $A\beta_{40}$ was co-incubated with or without SP1 (0.5-10µM) at 37°C for next 24h. After the incubation period 20µl of MTT reagent (5mg/ml in DPBS) was added to each well and incubated in a humidified atmosphere of 5% CO_2 at 37°C for 4h. Formazan crystals (purple) are formed by the metabolically active cells by cleaving the yellow tetrazolium salt. After the incubation period, the MTT solution was aspirated, and 150µL of dimethyl sulfoxide (DMSO) was added to each well to dissolve the formazan precipitate. Absorbance was measured at 570 nm with a reference filter at 690 nm by a microplate reader (Bio Rad, CA, USA). For time-dependent inhibition study, fresh $A\beta_{40}$ (10µM) was co-incubated with SP1 (5µM) upto 72h, and cell viability was measured. Results are expressed from three separate experiments as a percentage of controls.

c. LDH Release Assay

The release of cytosolic enzyme lactate dehydrogenase (LDH) into the culture medium indicates cell death due to the disruption of membrane integrity. Cell deathwas assessed by measuring LDH release in the culture medium by photocolorimetric kit according to the manufacturer's protocol (Abcam, Cambridge, MA, USA). An increase in the number of lysed cell increases LDH activity in the culture medium proportionately. In brief, SH-SY5Y cells were seeded for 12h into FBS-free medium and subsequently co-incubated with 10μM $A\beta_{40}$ at 37°C for 24h with or without different concentrations of SP1 (0.5, 1, 2.5, 5 and 10μM). Next, 50 µl culture supernatant was collected from each well and added to an assay mixture that was prepared by adding an equal amount of LDH substrate, co-factors, and dye solutions and incubated in the dark for 40mins at 37°C. Measurement of total LDH release was done by adding 1% (V/V) Triton X-100 into the cell for 1h. Absorbance was measured at 490 nm with a reference filter of 660nm. Percentage of (%) of total LDH activity (LDH in the supernatant + LDH in cell lysate) was used to express LDH leakage by the following formula:

$$\% \text{ LDH released} = (\text{LDH activity in the medium}/\text{total LDH activity}) \times 100$$

d. Measurement of intracellular ROS

Generation of intracellular reactive oxygen species was measured by a spectrofluorimetric method using peroxide sensitive fluorescent probe 2′,7′-Dichlorofluorescin diacetate (DCFH-DA).[11] In brief, SH-SY5Y cells were seeded in 96 well plate and incubated with 10μM $A\beta_{40}$ or SP1 at a concentration of 5μM alone or both for 24h. Next, DCFH-DA (Sigma-Aldrich, USA)was added in the medium and incubated for 40 min at 37°C in the dark. Intracellular esterases cleave non-fluorescent DCFH-DA into DCFH, which is oxidized to highly fluorescent DCF by intracellular ROS. Cells were then washed twice with PBS, and fluorescent intensity was measured by using a fluorescence microplate reader (Bio Rad, CA, USA) with an excitation filter of 490 nm and an emission filter of 520 nm.

e. Measurement of intracellular calcium (Ca^{2+})

Ca^{2+}sensitive dye Fura-2AM was used to measure free intracellular Ca^{2+}level in SH-SY5Y cell suspension by the previously described method.[12] Following treatment with the respective drug, Fura-2 AM (Cayman, USA) at a concentration of 5 μM was added to the culture medium and incubated at 37°C for 45 min. Next, Krebs-Ringer-HEPES (KRH) buffer (135mM NaCl, 5mM KCl, 1mM $MgSO_4$, 0.4mM KH_2PO_4, 1mM $CaCl_2$, 5.5mM glucose, 20mM HEPES, pH 7.4) was used to wash each plate twice containing (1×10^6cells). Fluorescent microplate reader (FLX 800, Biotek, USA) was used to measure the fluorescence intensity with an excitation and emission spectra of 488 and 525 nm, respectively. Results are Mean±SEM of quadruplicate measurements from three separate experiments and expressed in the increase in percent relative fluorescent unit (RFU) relative to the baseline reading.

f. Western Blot Analysis

Western blot analysis was performed to measure Bax, Bcl-2, cleaved Caspase 9 and cleaved Caspase 3, pro and anti-apoptotic proteins expression. About 5×10^6 cells were washed with PBS twice following treatment with SP1 and $A\beta_{40}$ for 24h with indicated dosage. Next the cells were homogenized with ice-cold lysis buffer (50mM Tris; pH 8.0, 150mM NaCl, 1% NP 40, 5mM EDTA; pH 8.0, 1% deoxycholic acid) that contained protease cocktail inhibitor (Sigma, St Louis, MO, USA). Electrophoresis of respective proteins was done on 15% (W/V) SDS- Polyacrylamide gel (MiniPROTEAN® tetra cell with mini-trans Blot®, Bio-Rad, USA) which was then transferred onto a nitrocellulose membrane (Millipore, USA). Next, incubation of the membrane was done with fresh blocking buffer (10 mm Tris–HCl, pH 8.0, 150 mm NaCl, 0.05% Tween-20 containing 5% nonfat-dried milk) for 1h at room temperature and then probed with mouse primary monoclonal anti-Bax(Santa Cruz Biotechnology, Inc., USA; 1:800 dilution in 3% BSA), mouse anti-Bcl-2 (Abcam, MA, USA; 1:1000 dilution in 3% BSA), polyclonal rabbit anti cleaved caspase-9 (Abcam, Cambridge, MA, USA; 1:1000 dilution in 3% BSA), monoclonal rabbit anti cleaved caspase-3 (Abcam, Cambridge, MA, USA; 1:700 dilution in 3% BSA) respectively at 4°C

overnight. Then the membranes were washed three times with TBST solution followed by incubation with HRP conjugated goat anti-mouse (Santa Cruz Biotechnology, Inc., USA; 1:5000 dilution in 3% BSA), goat anti-rabbit (Abcam, Cambridge, MA, USA; 1:5000 dilution in 3% BSA) secondary antibody for 2h at room temperature. Then the membranes were washed three times in the TBST buffer and stripped by stripping solution. To reduce internal blot variability, incubation was done with anti-β-actin rabbit polyclonal antibody (Abcam, Cambridge, MA, USA; 1:5000 dilutions in 3% BSA). ECL substrate solution (Thermo Scientific, Rockford, USA) was used to visualize immunoreactive bands. The optical density (OD) value of each band was analyzed by the electrophoresis image analysis system (Smartview 2001, S/N: SV-0002202, Japan) and normalized against the corresponding β-actin band.

1.4. Methods for *in vivo* animal-based experiments

a. Animals

Male Sprague–Dawley rats weighing 220-250 g were obtained from Chittaranjan National Cancer Institute (CNCI), Kolkata, India. Before the experimentation, all animals were allowed to acclimatize at least one week on their home cages at 21±2°C temperature with 55% humidity under constant 12 h light/dark cycle (lights on at 6:00 a.m., lights off at 6:00 p.m.) with food and water *ad libitum.* All experiments conformed to the guidelines of the Committee for the Purpose of Control and Supervision of Experiments on Animal (CPCSEA, New Delhi). The experimental protocols were approved by the Institutional Animal Ethics Committee (IAEC) of Raja Peary Mohan College, Uttarpara, Hooghly, West Bengal (Reg. No. 1148/PO/ac/CPCSEA). The whole experiment was divided into two parts to fulfill key objectives.

b. Design of experiments

24 male Sprague Dawley rats were randomly divided into three groups: **control**, **SP1**A, and **SP1**B. Each group contains 8 animals and administration of **SP1**was performed for 42 days through a tail vein by the following schedule: ***Control***: *Without any treatment.*

SP1*A: Intravenous injection of 100 µg/kg body wt. of* **SP1** *into the tail vein.*

SP1*B: Intravenous injection of 500 µg/kg body wt. of* **SP1** *into the tail vein.*

Rats were sacrificed after 42 days of injection schedule and blood was collected for hematological and biochemical estimations. For the histopathological study, the liver and kidney were also isolated very carefully and checked for cytoarchitectural changes if any.

c. Preparation of SP1 and Administration of SP1 into the tail vein

The body weights and volumes of **SP1** to be administered were recorded for each animal. Prior to injection, the tail of the animal was warmed by using warm water circulating pad placed under the cage for 5-10 minutes to dilate the veins. A 25-gauge needle was inserted into the vein towards the direction of the head. The needle and syringe were kept parallel to the tail. Now the respective dosage of **SP1 (**100 µg/kg and 500 µg/kg) was injected into the tail slowly. The needle was removed very carefully and gentle compression applied until bleeding was stopped. Animals were returned to their home cage.

d. Analysis of blood for hematological and biochemical parameters in respect to SP1 toxicity

The biochemical parameters including blood glucose, serum urea, creatinine, total bilirubin, serum glutamic oxaloacetic transaminase (SGOT), serum glutamic pyruvic transaminase (SGPT), and alkaline phosphatase (ALP) were analyzed for sub-chronic toxicity of **SP1**by using commercially available diagnostic test kits (Crest Biosystems kits, India). Blood parameters like red blood cells, white blood cells, hemoglobin, neutrophil, lymphocyte, eosinophil, monocyte, and basophil were estimated using standard kits by following the manufacturer's manual.

e. Histopathological experiments for sub-chronic toxicity

Histopathological analysis was carried out on the preserved liver and kidney tissues. The **SP1** treated rats and control rats were sacrificed by decapitation after 42 days of treatment schedule. Organs were removed and fixed in 10% buffered neutral formalin for 48 h. The fixed organs were embedded in paraffin wax. Sections were cut by using a microtome with 5-micrometer thickness. Slides were stained with Haematoxylin and eosin (H &E staining) for histological examination. Slides were observed under the light microscope (Dewinter Smart, India)for histopathological changes, and images were taken at 40×magnification.

f. Preparation of stock toxic Aβ_{40} and SP1

The lyophilized Aβ_{40} peptide was dissolved in artificial cerebrospinal fluid (aCSF: 119mM NaCl, 26.2mm $NaHCO_3$, 2.5mM KCl, 1mM NaH_2PO_4, 1.3mM $MgCl_2$, 10mM glucose; pH 7.4) solution at a concentration of 1mg/ml for stock and incubated at 37°C for 72 h to induce aggregation. The solution finally kept at 4°C before using it. **SP1** was also dissolved in aCSF with a concentration of 1mM/L and respective concentrations of **SP1**were prepared from this stock solution.

g. Intra-hippocampal microinjection of toxic Aβ_{40} and SP1by stereotaxic surgery

Thirty male Sprague-Dawley rats were randomly divided into 5 groups, and each group contains six animals (n = 6). They were as following:

Group 1: Control (No Treatment)

Group 2: Sham (Bilaterally microinjection of aCSF into the hippocampus)

Group 3: Aβ_{40} (Bilaterally microinjection of toxic Aβ_{40} into the hippocampus)

Group 4: **SP1** (40µM) + Aβ_{40} (Bilaterally microinjection of **SP1** and Aβ_{40} into the hippocampus)

Group 5: **SP1** (100µM) + Aβ_{40} (Bilaterally microinjection of **SP1** and Aβ_{40} into the hippocampus).

The whole experimental procedure was designed for 28 days. From the aforementioned groups, each rat was habituated in the laboratory environment for the first five days of 28 days schedule. On the 6th day, all rats from the aforementioned groups (except control group) were placed in the stereotaxic platform for surgery and toxic $A\beta_{40}$ bilaterally infused into the hippocampus. All rats were kept their home cage for recovery of 7 days. **SP1** was micro infused bilaterally into the hippocampus after seven days from recovery, i.e., from 14th days to 28th days of experimental schedule. All animals were sacrificed after 28th days, and brains were isolated and kept for experimental analysis.

h. Stereotaxic Surgery

Rats were anesthetized by intraperitoneal injection of a combination of Ketamine hydrochloride (80 mg/kg body wt) and Xylazine hydrochloride (10 mg/kg body wt.).[13]The surgical site was shaved to expose the skin and cleaned with betadine lotion. The scalp was incised roughly 1.5 mm in length to expose the sagittal suture, bregma, and lambda of the skull. Two small holes were made on the corresponding positions by 0.8mm drill head according to the predetermined stereotaxic coordinates (anteroposterior: -3.8 mm from Bregma, medial/lateral: ±2.2 mm and dorsoventral: -2.7 mm).[14,15] Guide cannula (0.50mm diameter) was placed for the intra-hippocampal microinjection into the corresponding position. The guide cannula was anchored to the skull with two adjacent stainless-steel screws andacrylic dental cement. To prevent occlusion and infection, a dummy cannula was inserted over the guide cannula. All rats were allowed a minimum of seven days to recover from surgery. 3 µL of 20µM/ml toxic $A\beta_{40}$ was injected bilaterally into the hippocampus of $A\beta_{40}$ group at a flow rate of 1 µL/ min using stainless steel cannula (0.55 mm diameter) connected to a Hamilton syringe at the day of surgery (single bolus). Rats of the sham-operated group received the same volume (3 µL) of artificial CSF in each lateral ventricle by the same procedure.A volume of 3 µL of 40µM/ml and 100µM/ml of **SP1** solution injected into the hippocampus of the **SP1**-treated group daily for 28 days as above mentioned procedure after seven days of recovery from surgery. The injection cannula was left in place for 2 min after injection to ensure the complete infusion of the drug. The Control group of rats has received no treatments.

i. Cresyl violet staining of hippocampal neurons to assess neuroprotective effects of SP1:

The sections were deparaffinised in xylene with two changes at 10 minutes each and dehydrated with 100% alcohol (2x3 min.), 95% alcohol (2 min.), 70% (2 min.). Warm Cresyl fast violet staining solution (0.1g %) was allowed to stain the section for 20-30 minutes for better impregnation. After rinsing the section with distilled water, it was treated with 96% alcohol and absolute alcohol. The section was mounted in DPX after clearing with xylene, 3x5 min. The intensity of Nissl granules was measured using Image J Launcher software (v1.4.3.67; NIH, Bethesda, MD). A total of six microscopic fields/brain areas were examined per rat. The mean value of the intensity of Nissl granules present/field of brain area/animal or field was calculated from the six rats/ regimen.

2. List of all the peptides and their characterization data

Supplementary Table 1. Sequences of the synthesized peptides with their observed functions

Peptides	Sequences	Functions
**SP1**	H$_2$N-A**(*N*-Me)F**F**(*N*-Me)V**LG-Succinyl-PEG$_3$-Adipoyl-G**(*N*-Me)L**V**(*N*-Me)F**FA-NH$_2$	Inhibitor
**CBp**	Ac-G**(*N*-Me)L**V**(*N*-Me)F**FA-NH$_2$	Inhibitor/control
**SP1A**	H$_2$N-A**(*N*-Me)F**F**(*N*-Me)V**LG-Succinyl-PEG$_3$-Adipoyl-G**(*N*-Me)L**V**(*N*-Me)F**FAW-NH$_2$	FRET donor (D)
**SP1B**	H$_2$N-XA**(*N*-Me)F**F**(*N*-Me)V**LG-Succinyl-PEG$_3$-Adipoyl-G**(*N*-Me)L**V**(*N*-Me)F**FA-NH$_2$	FRET acceptor (A)
**SP1C**	H$_2$N-XA**(*N*-Me)F**F**(*N*-Me)V**LG-Succinyl-PEG$_3$-Adipoyl-G**(*N*-Me)L**V**(*N*-Me)F**FAW-NH$_2$	FRET donor-acceptor (D/A)
**LP1A**	GLVFFAW-NH$_2$	FRET donor (D)
**LP1B**	GLVFFAX-NH$_2$	FRET acceptor (A)

Note: (X=2-amino-3-(5-(dimethylamino)naphthalene-2-sulfonamido)propanoic acid, (dansyl)), W=Tryptophan, SP=Synthetic Paratope, LP= Linear Peptide. **SP1A**, **SP1B**, **SP1C**, **LP1A** and **LP1B** represent fluorophore attached species and were synthesized for FRET study. N-methylation was represented by bold black letters in peptide sequences.

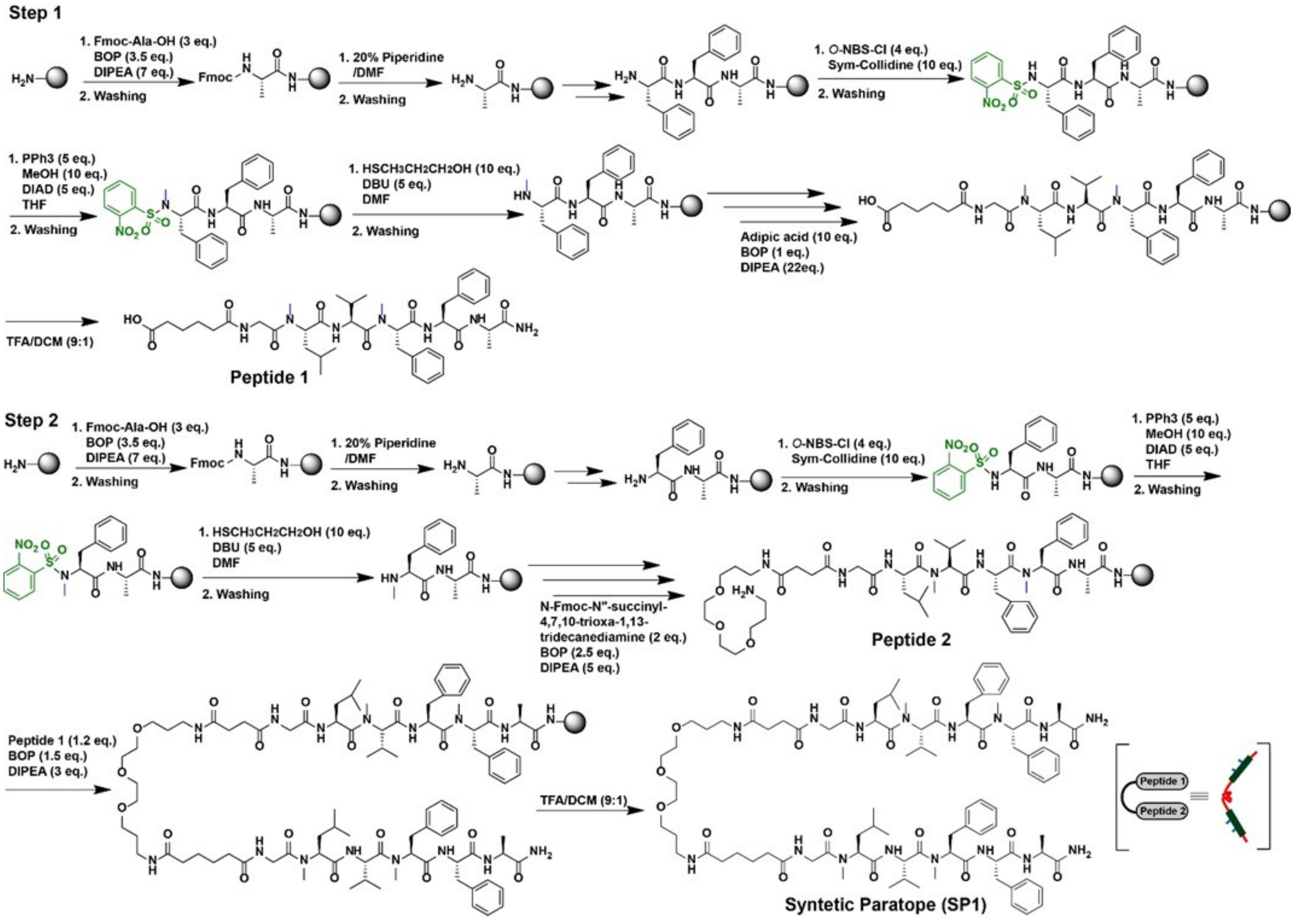

Supplementary Scheme 1. Schematic presentation of the synthesis of the synthetic paratope (**SP1**).

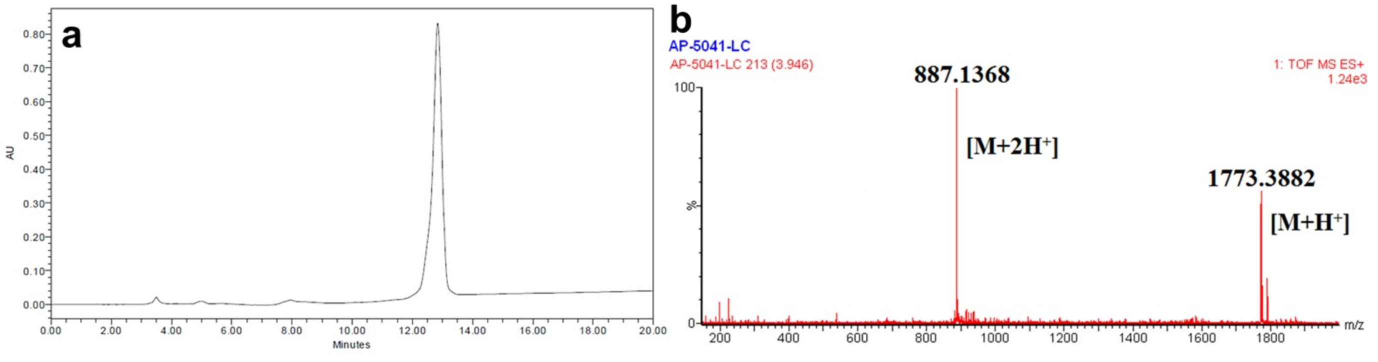

Supplementary Figure 1.(a) HPLC profile of the purified synthetic paratope (**SP1**). (b) Mass spectrum of synthetic paratope (**SP1**). Calculated mass for $C_{92}H_{139}N_{16}O_{19}$ is 1773.04 $[M+H]^+$, observed 1773.38 $[M+H]^+$ and 887.1368 $[M+2H]^{2+}$.

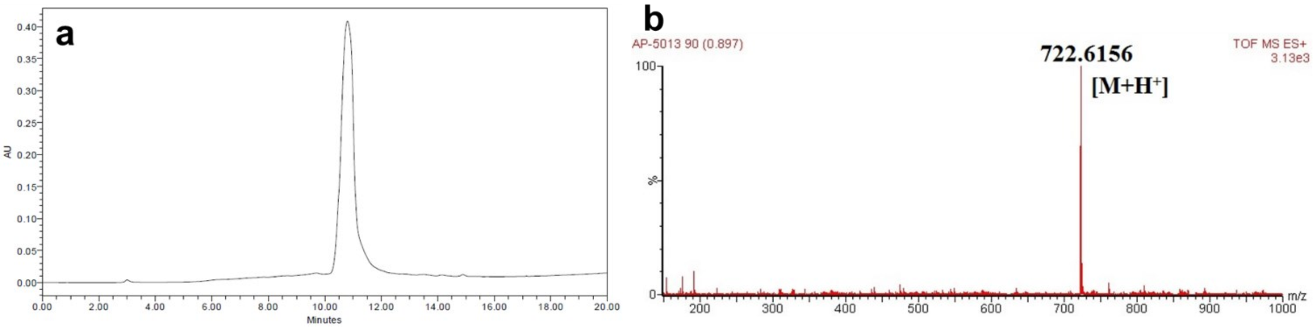

Supplementary Figure 2. (a) HPLC profile of the purified control breaker peptide (**CBp**). (b) Mass spectrum of the control breaker peptide (**CBp**). Calculated mass for $C_{38}H_{56}N_7O_7$ is 722.42 $[M+H]^+$, observed.

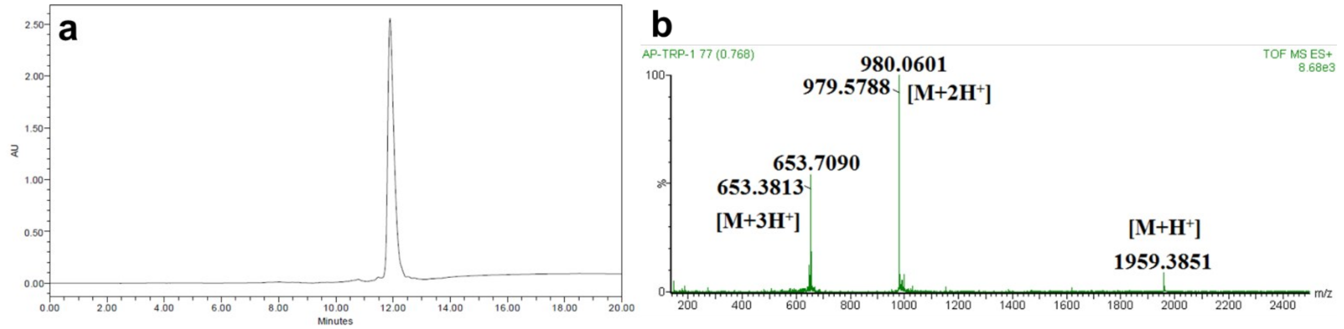

Supplementary Figure 3. (a) HPLC profile of the purified peptide **SP1A**. (b) Mass spectrum of **SP1A**. Calculated mass for $C_{103}H_{149}N_{18}O_{20}$ is 1959.12 $[M+H]^+$, observed 1259.38 $[M+H]^+$, 980.06 $[M+ 2H]^{2+}$ and 653.71 $[M+3H]^{3+}$.

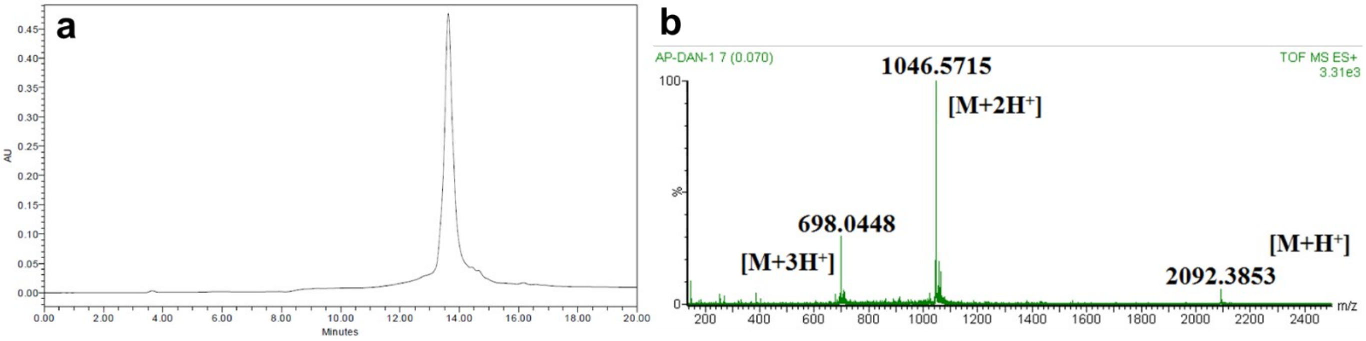

Supplementary Figure 4. (a) HPLC profile of the purified **SP1B**. (b) Mass spectrum of **SP1B**. Calculated mass for $C_{107}H_{156}N_{19}O_{22}S$ is 2092.14 $[M+H]^+$, observed 2092.38 $[M+H]^+$, 1046.57 $[M+2H]^{2+}$ and 698.04$[M+3H]^{3+}$.

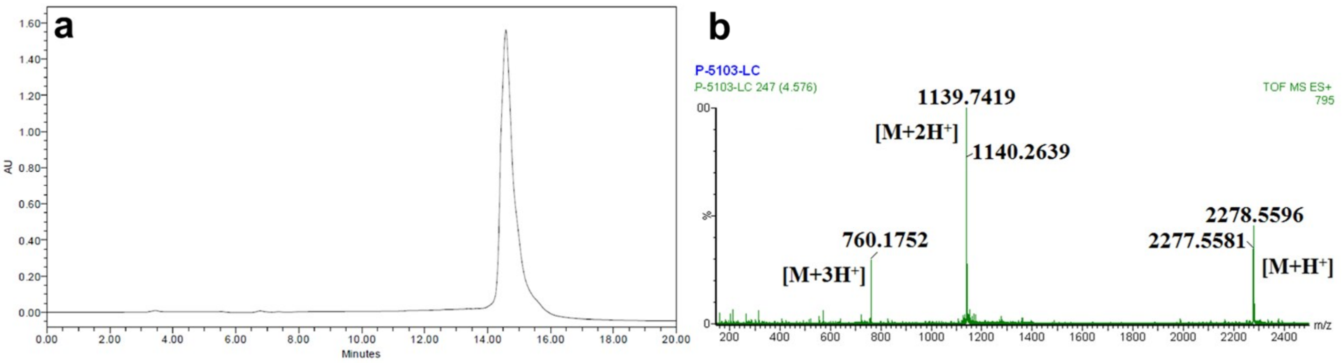

Supplementary Figure5. (a) HPLC profile of the purified **SP1C**.(b) Mass spectrum of **SP1C**. Calculated mass for $C_{118}H_{166}N_{21}O_{23}S$ is 2278.22 $[M+H]^{+}$, observed 2278.55 $[M+H]^{+}$, 1139.74 $[M+2H]^{2+}$ and 760.17 $[M+3H]^{3+}$.

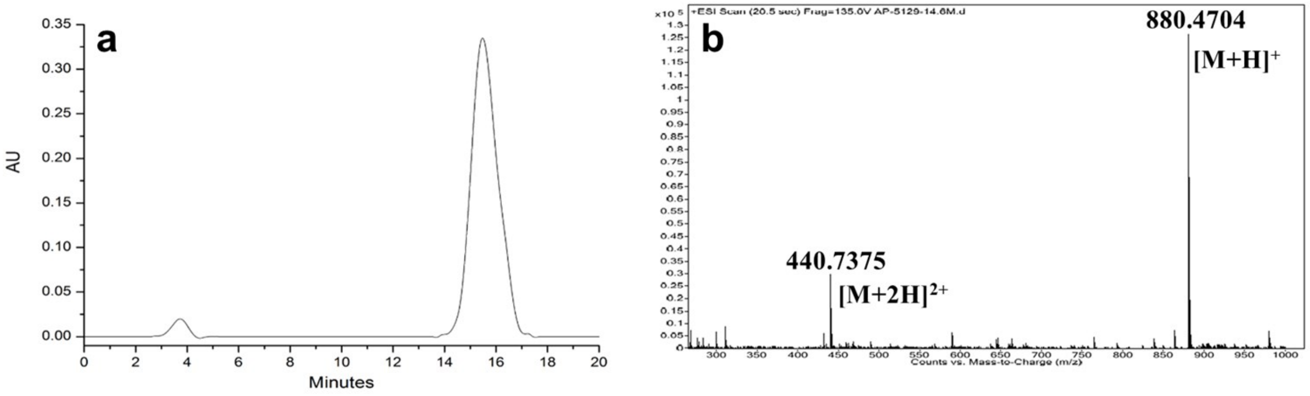

Supplementary Figure 6. (a) HPLC profile of the purified LP1A. (b) Mass spectrum of **LP1A**. Calculated mass for $C_{47}H_{61}N_{9}O_{8}$ is 880.4677 $[M+H]^{+}$, observed 880.4704 $[M+H]^{+}$, 440.7375 $[M+2H]^{2+}$.

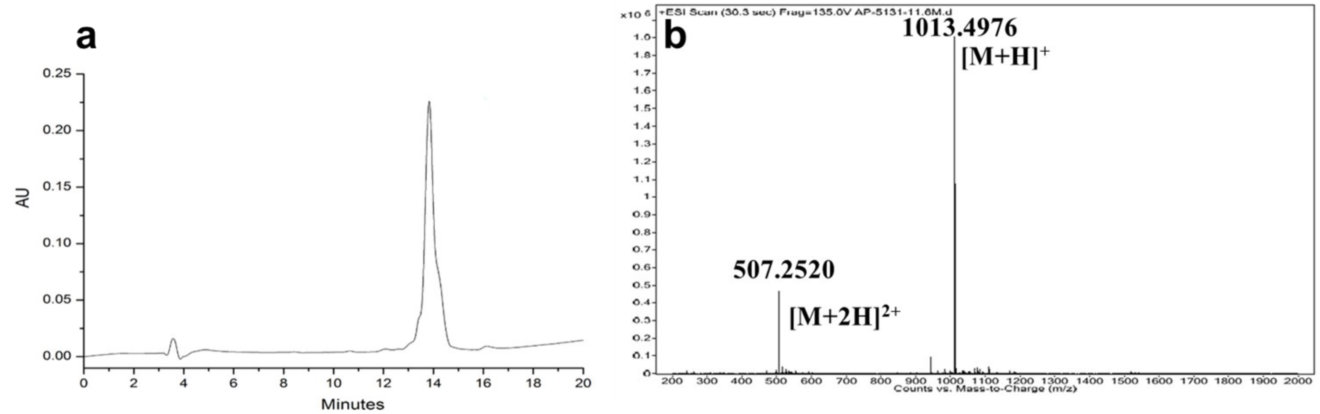

Supplementary Figure7. (a) HPLC profile of the purifiedLP1B.(b)Mass spectrum of **LP1B**, calculated mass for $C_{51}H_{68}N_{10}O_{10}S$ is 1013.4874 $[M+H]^{+}$, observed 1013.4976 $[M+H]^{+}$, 507.2520 $[M+2H]^{2+}$.

3. Additional spectra and figures

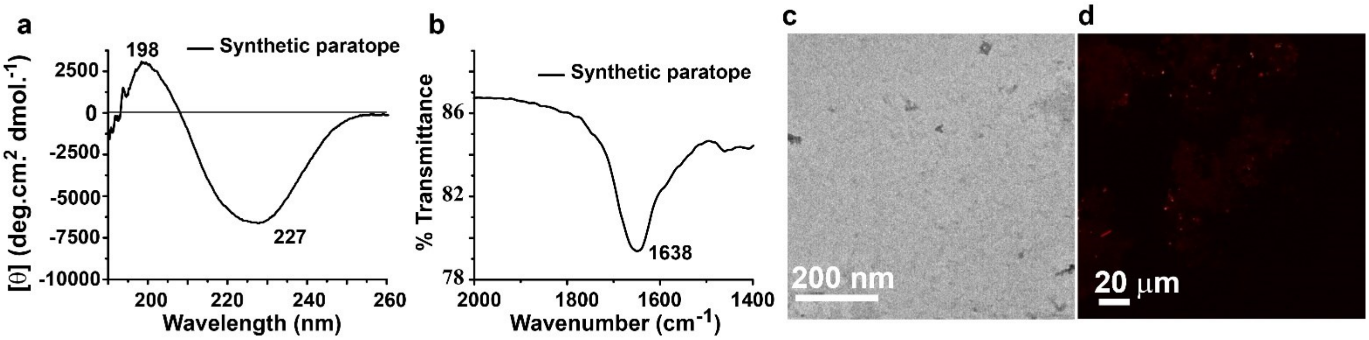

Supplementary Figure 8. (a) CD and (b) FTIR spectrum of the **SP1.** (c) TEM and (d) Congo red birefringence images of the **SP1**. The concentration of the **SP1** for the analysis was 100μM in PBS pH 7.4 (in the presence of 3% DMSO).

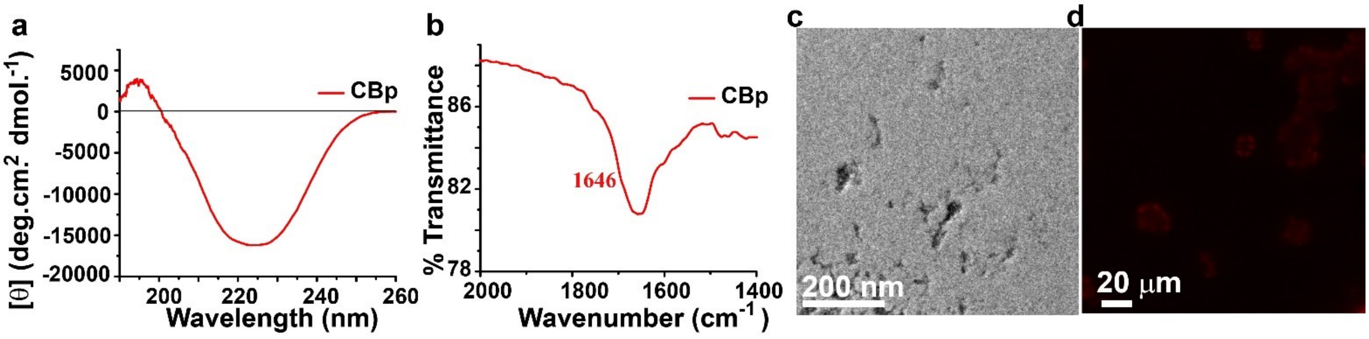

Supplementary Figure 9. (a) CD and (b) FTIR spectrum of the **CBp.** (c) TEM and (d) Congo red birefringence images of the**CBp**. The concentration of the SP1 for the analysis was 100μM in PBS pH 7.4 (in the presence of 3% DMSO).

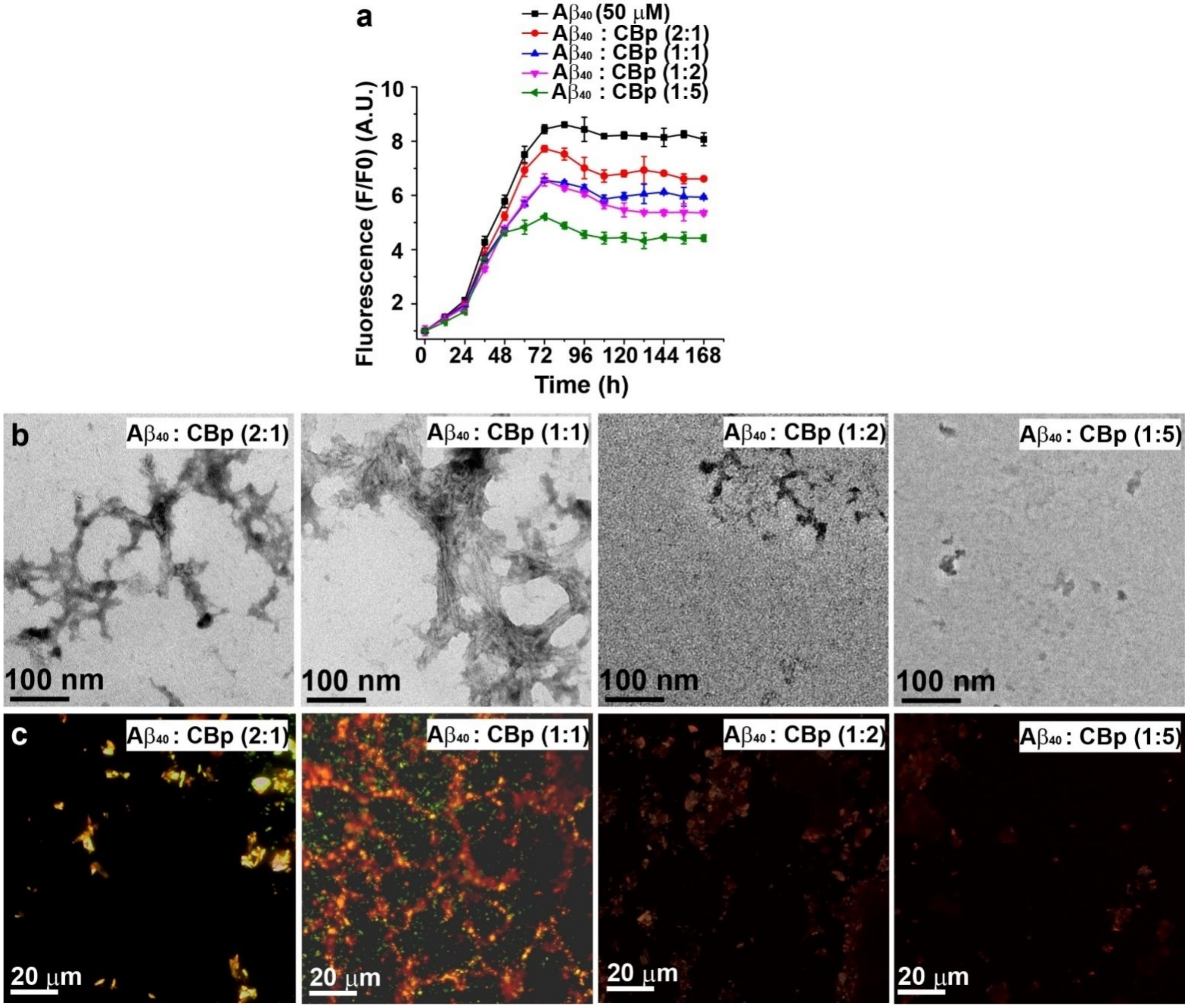

Supplementary Figure 10. (a) ThT assay,(b) TEM, and (c) Congo red birefringence images of Aβ_{40} in the presence of 0.5-fold (i), 1-fold (ii), 2-fold (iii) and 5-fold (iv) molar excess of the**CBp**. Experiments were performed in PBS of pH 7.4 at 37 °C (in the presence of 3% DMSO).

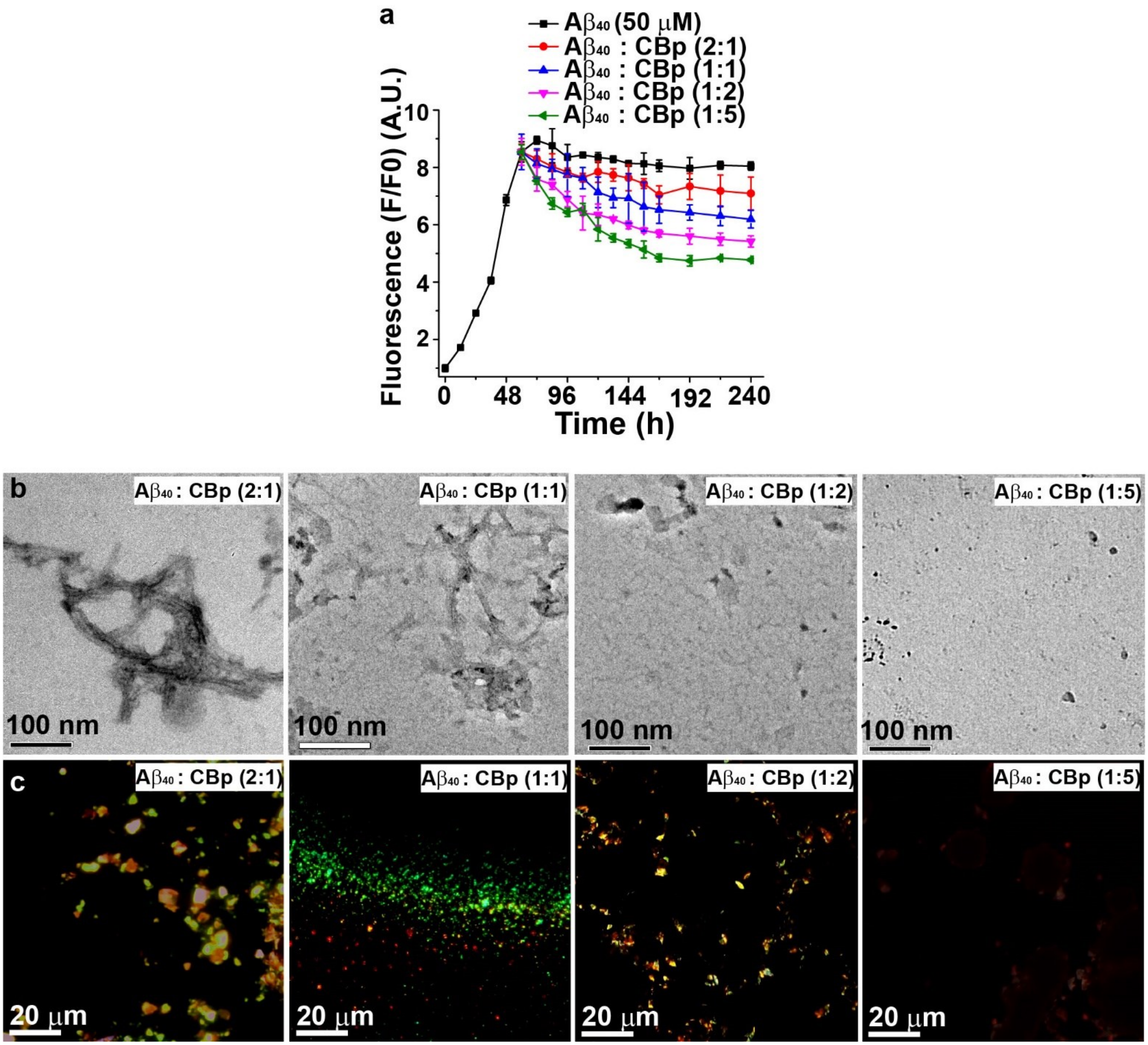

Supplementary Figure 11. (a) ThT assay, (b) CD, and (b) FTIR spectra, for the disaggregation of the preformed fibrils of Aβ_{40} in the absence (black) or presence of 0.5-fold (red), 1-fold (blue), 2-fold (magenta) and 5-fold (olive) molar excess of **CBp.** (d) TEM and (e) Congo red birefringence images of Aβ_{40} in the presence of 0.5-fold (i), 1-fold (ii), 2-fold (iii), and 5-fold (iv) molar excess of the **CBP**. Experiments were performed in PBS of pH 7.4 at 37 °C (in the presence of 3% DMSO).

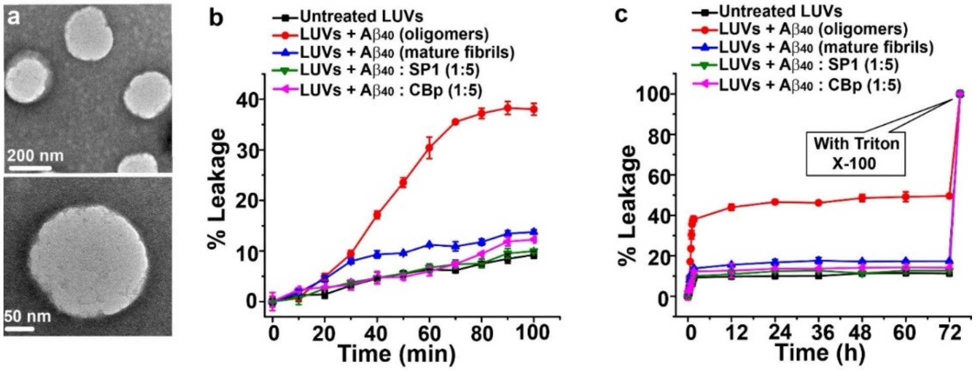

Supplementary Figure 12. (a) TEM images of dye loaded LUVs at a concentration of 100 µM of lipids in 50 mM HEPES (stock of 2 mM). The spectra showing % of dye leakage by untreated LUVs (black); LUVs treated with Aβ_{40}oligomers (red), Aβ_{40}mature fibrils (blue), disrupted Aβ_{40}fibrils by **SP1** (olive) or **CBP**(magenta) from time 0 to 100 min (b) and 0 to 72h (c).

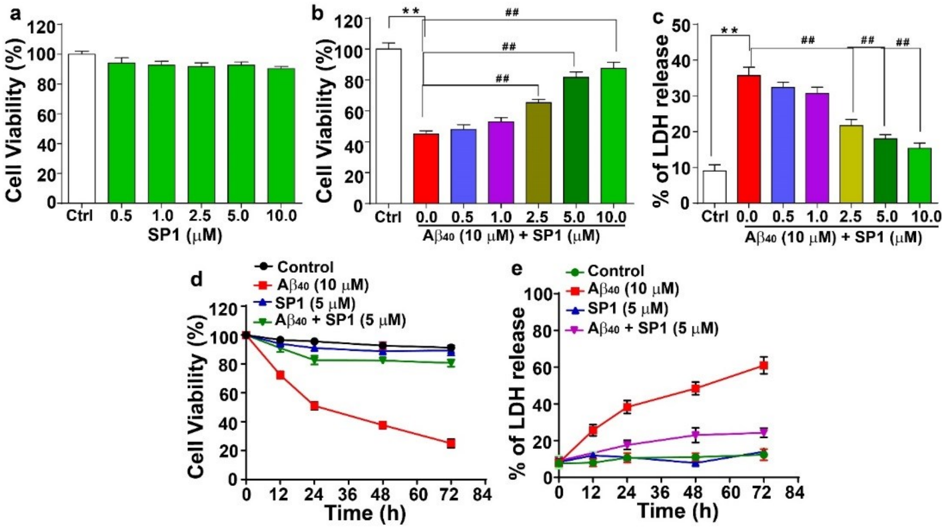

Supplementary Figure 13. Amelioration of Aβ40 induced cytotoxicity by the SP1. (a)To study the self-toxicity of **SP1** on cell viability of human neuroblastoma SH-SY5Y cells. Cells were incubated with different concentrations (0.5-10 µM) of **SP1** for 24h. (b) Aβ_{40} monomer (10 µM) was incubated in the absence or presence of a different concentration of the **SP1**(0.5-10 µM) for 24h at 37°C. Cell viability was determined by MTT assay. (c) After 24h of treatment with Aβ_{40} (10 µM) in the absence or presence of the **SP1** (0.5-10 µM), cytotoxicity was measured by % of LDH release from the damaged cell membrane. (d) Time course effect of the **SP1** (5 µM) inhibition of Aβ_{40} (10 µM) induced neurotoxicity. (e) Time-dependent kinetics study of **SP1** on inhibition of Aβ_{40} (10µM) induced LDH leakage into the medium. Cells that stain green are alive, and those stained orange are dead. Values are represented as Mean ± SEM (n=3 separate experiments). **p<0.01, compared to the control group and ##p<0.01 compared to Aβ40 treated group, One-way ANOVA.

Supplementary Table 2. Original Blots of each Western Blot analysis (Fig. 3e)

**Different Protein expression**	**Control** **SP1** **$A\beta_{40}$** **$A\beta_{40}$+SP1**
**Bax**	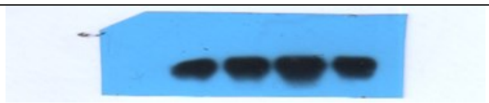
**Bcl-2**	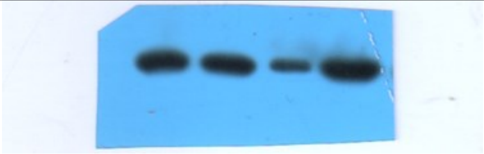
**Cleaved Caspase-9**	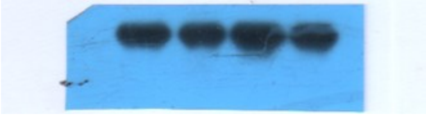
**Cleaved Caspase-3**	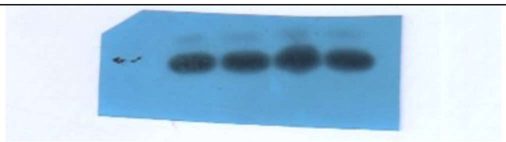
**β-actin**	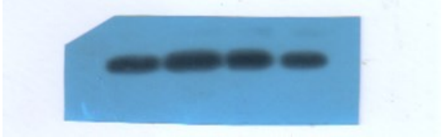

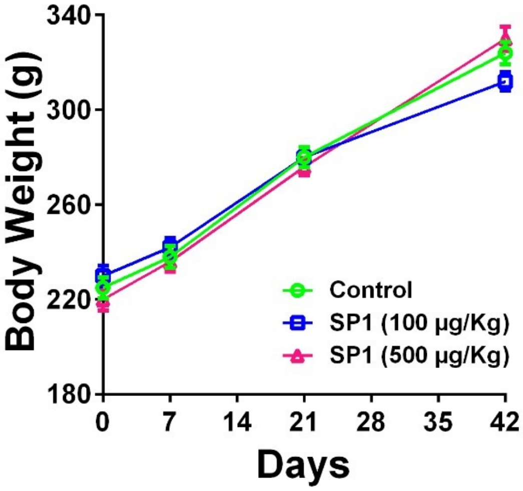

Supplementary Figure 14. Effect of **SP1** on the bodyweight of rats (n= 8 in each group) in sub-chronic toxicity study.

Supplementary Table 3. Effect of **SP1** on hematological and biochemical parameters in rats (n= 8 in each group).

	Control Rats	**SP1**treated rats	
		100µg/Kg	500µg/Kg
Hematological Parameters			
RBC ($10^6/mm^3$)	4.77±1.54	5.20±1.23	5.51±1.38
WBC ($10^3/mm^3$)	6.60±1.20	5.80±1.34	6.21±1.15
Hb (%)	12.00±2.47	13.6±2.62	13.2±0.95
Neutrophil (%)	56.6±2.31	58.00±2.54	62.10±2.42
Lymphocyte (%)	38.2±3.25	42.3±2.92	40.5±3.12
Eosinophil (%)	3.12±0.61	2.62±0.82	2.72±0.53
Monocyte (%)	3.20±0.99	3.8±1.08	5.20±1.24*
Basophil (%)	0.00±0.00	0.00±0.00	0.00±0.00
Biochemical Parameters			
Glucose (mg/dl)	103.0±1.34	110.0±1.36	105.0±1.28
Serum Urea (mg/dl)	26±1.45	32.0±1.83	29.0±1.66
Creatinine (mg/dl)	0.73±0.02	0.69±0.05	0.86±0.03
Total Bilirubin (mg/dl)	0.58±0.04	0.54±0.09	0.72±0.06
SGOT (IU/L)	52.0±2.21	55.0±2.94	64.2±2.56*
SGPT (IU/L)	38.0±1.70	44.0±1.84	40.32±1.63
ALP (IU/L)	111.0±3.46	120.0±3.68	116.0±2.15

RBC, red blood cells; WBC, white blood cells; Hb, hemoglobin; SGOT, serum glutamic oxaloacetic transaminase; SGPT, serum glutamic pyruvic transaminase; ALP, alkaline phosphatase. *P<0.01 as compared to control.

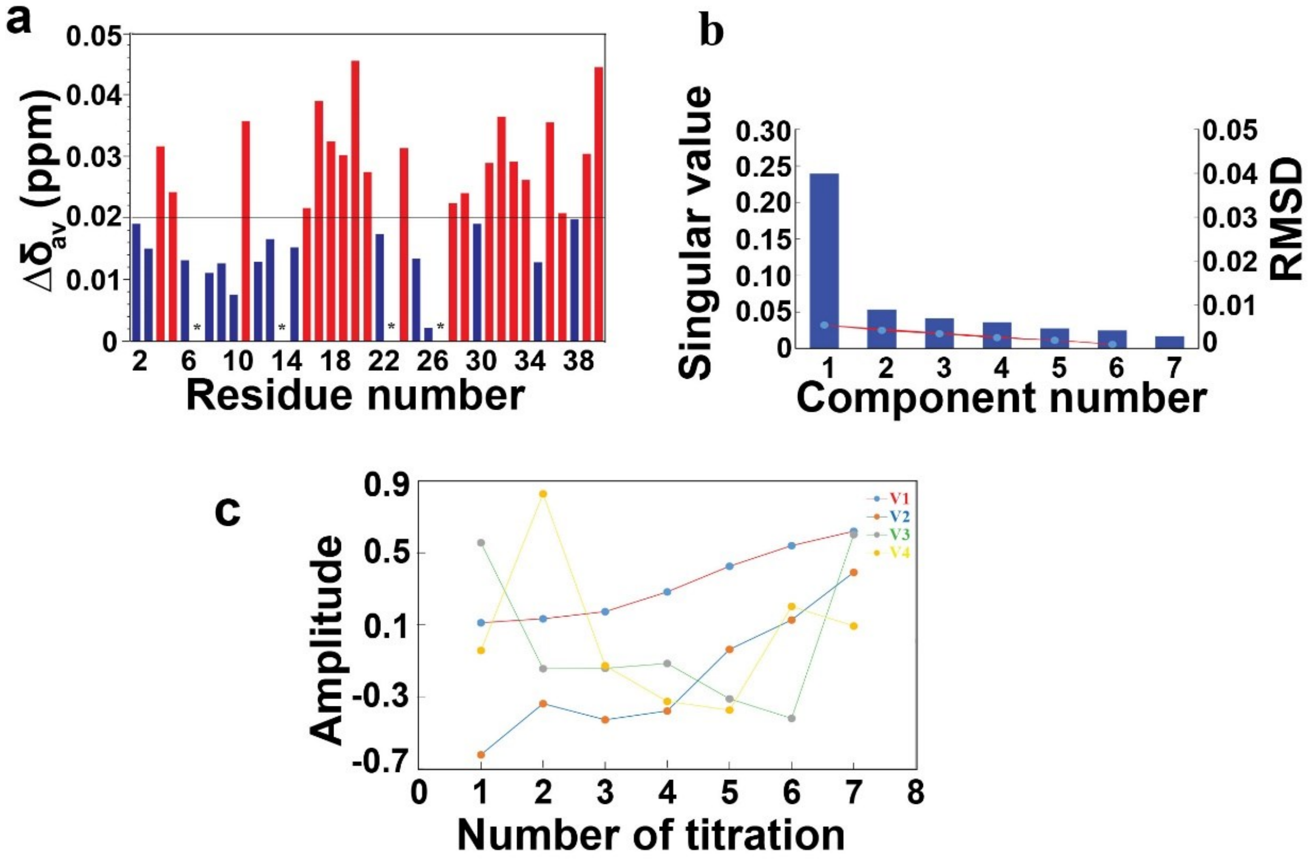

Supplementary Figure 15. a, Bar plot of CSP (Aβ_{40}: **SP1**=1:10) Aβ40 due to binding of **SP1**. The black threshold line represents the mean of all CSP values. Red bars indicate most interacting residues. Overlapping residues with similar chemical shifts are marked with an asterisk (*). **b**. SVD analysis of ^{15}N -Aβ 40 titrations with **SP1** (unlabeled). Blue bars are indicating singular values in descending order. The solid red circles indicate root mean square deviation (RMSD) between raw dataset and reconstructed dataset. Reconstructed dataset having all components results in the RMSD of 0, which is not shown in the figure. **c**, The first four components of v_i vectors are plotted against the number of titrations. The first two components have a smooth shape (red and blue lines).

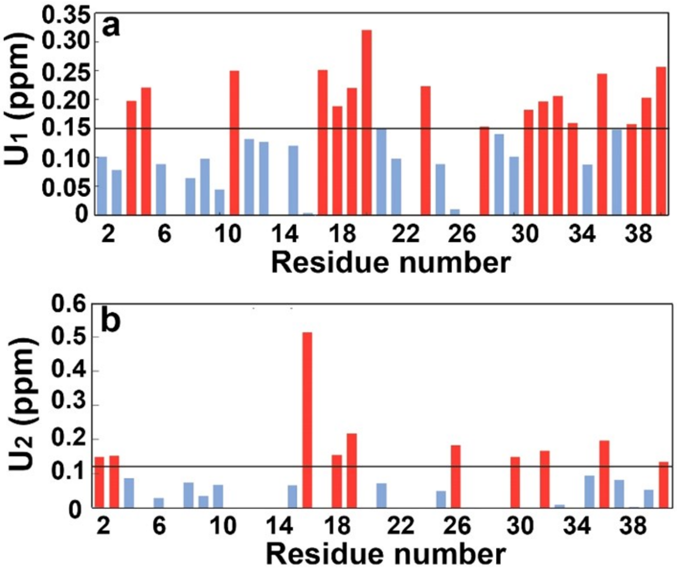

Supplementary Figure16. Bar plot of (a) first left singular vector, U_1 (b) second singular vector, U_2 plotted against residue number. Overlapping residues or residues with negative values are not shown in the figure.

Supplementary Table 4. Equilibrium dissociation constant calculated both before and after SVD.

Residue	$K_D$beforeSVD	K_D after SVD
R5	99.84	183.1
L17	336.5	132.4
F20	301.3	398
E22	961.2	419.9
V24	247.6	282.5
K28	196.6	180
G29	446.8	236.7
I31	396.2	398.2
G33	376.2	583
L34	435	385.6
G38	336	472.2

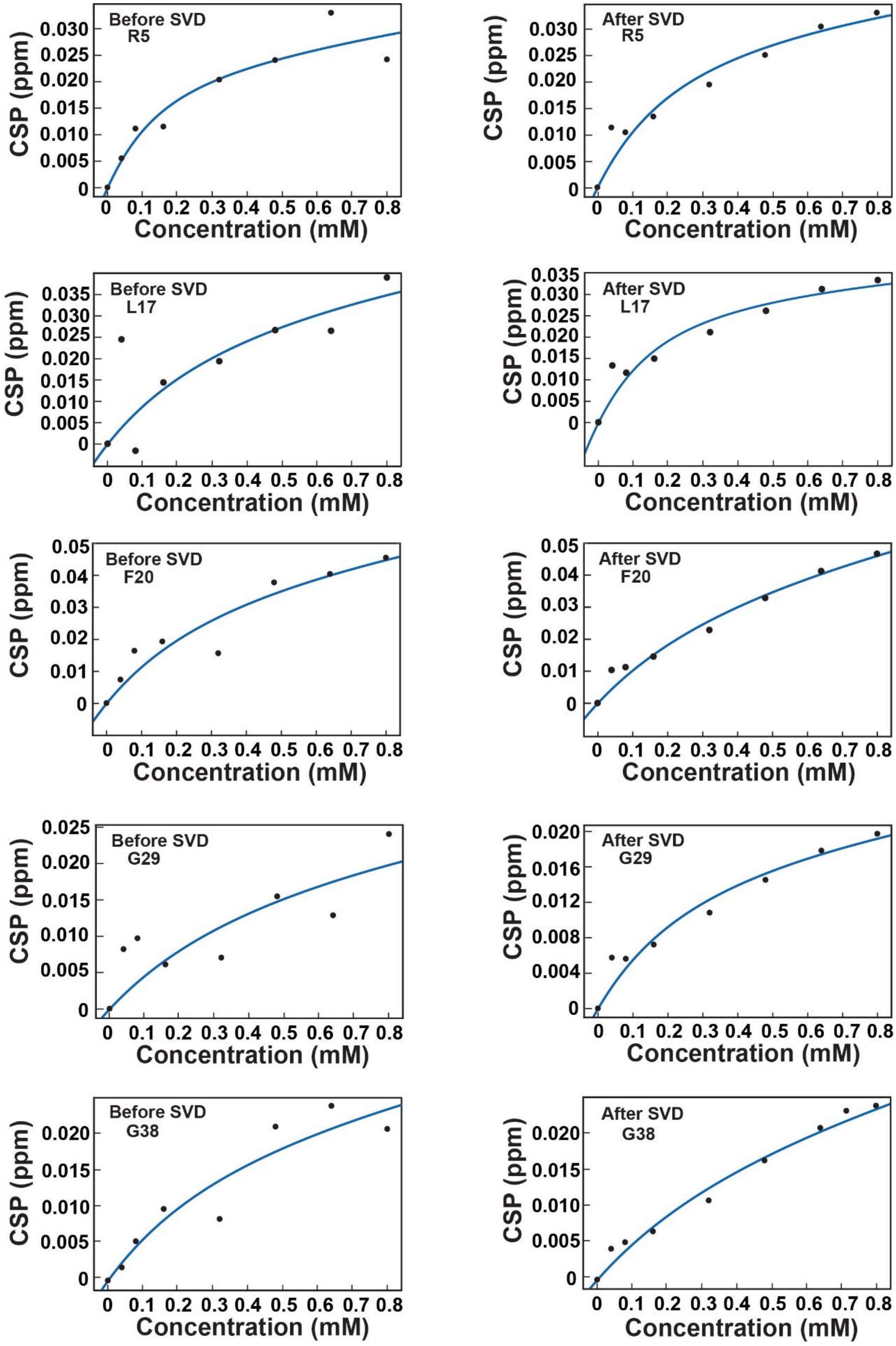

Supplementary Figure 17. Global fitting of the titration curves before SVD (left side) and after SVD (right side) reconstruction using only two non-noise components.

Supplementary Table 5. Summary of the fluorescence lifetimes of the **SP1A**, **SP1B**, **SP1C,**and the mixture (**SP1A**+**SP1B**). All the peptides were excited at λ_{ex}= 290 nm; Concentration of all the peptides were same = 20μM; <τ>, k_f and k_{nr} are weighted means from the biexponential fits: $<\tau> = 1/(\alpha_1/\tau_1 + \alpha_2/\tau_2)$, $k_f = \Phi/<\tau>$, and $k_{nr} = (1-\Phi_f)/<\tau>$.[9]

peptide	Φ_f	λ_{em} (nm)	τ_1 (ns)	τ_2 (ns)	< τ > (ns)	k_f (10^8 s^{-1})	k_{nr} (10^8 s^{-1})	χ^2
**SP1A**	0.06	355	1.352 (27%)	3.559 (73%)	2.951	0.202	3.186	0.98
**SP1B**	0.051	495	3.124 (48%)	8.741 (52%)	6.033	0.085	1.573	1.02
**SP1C**	0.058	355	1.069 (70%)	3.345 (30%)	1.772	0.328	5.315	0.98
	0.058	495	6.260 (15 %)	16.728 (85 %)	15.145	0.038	0.622	1.03
Mixture peptide (**SP1A**+**SP1B**)	0.65	355	1.350 (28%)	3.500 (72%)	2.902	0.222	0.3224	1.03
	0.65	495	3.857 (43%)	6.750 (57%)	5.486	0.118	1.705	0.98

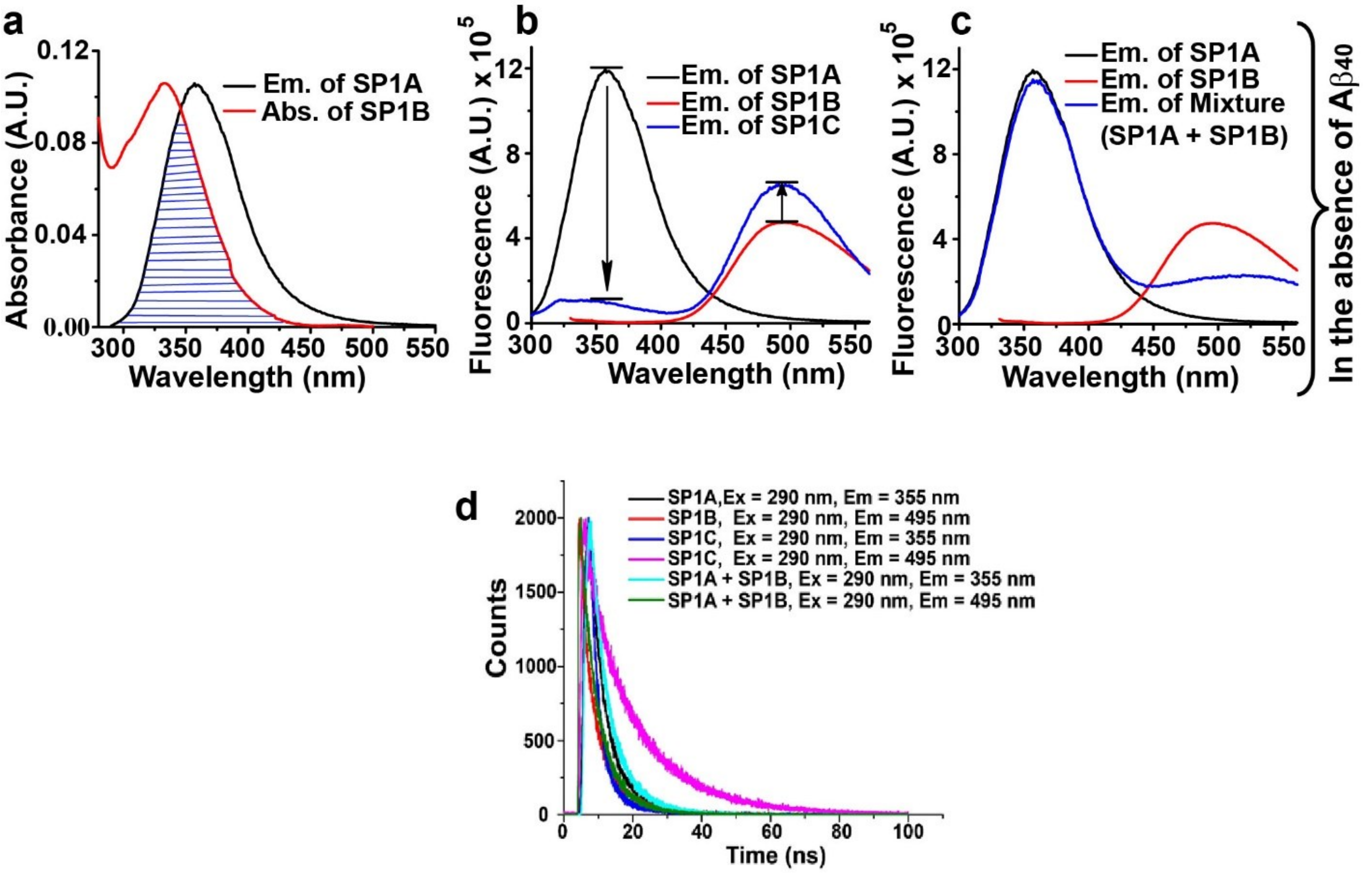

Supplementary Figure 18. a. Overlap of the emission spectrum of the donor, **SP1A** (black), and the absorbance spectrum of the acceptor, **SP1B** (red). **b.** Emission spectra of **SP1A** (black, Ex=290 nm), **SP1B** (red, Ex=320 nm) and **SP1C** (blue, Ex=290 nm). **c**. Emission spectra of **SP1A** (black, Ex=290 nm), **SP1B** (red, Ex=320 nm), and mixture (**SP1A+SP1B**, blue, Ex=290 nm). **d.** Time resolved fluorescence spectra of the peptide **SP1A** (black, λ_{ex} = 290 nm, λ_{em} = 355 nm), **SP1B** (red, λ_{ex} = 290 nm, λ_{em} = 495 nm), **SP1C** (for blue, λ_{ex} = 290 nm, λ_{em} = 355 nm and for magenta, λ_{ex} = 290 nm, λ_{em} = 495 nm) and mixture, **SP1AC**+**SP1B** (for cyan, λ_{ex} = 290 nm, λ_{em} = 355 nm and for olive, λ_{ex} = 290 nm, λ_{em} = 495 nm). Spectra were recorded in 20μM of peptide solutions in PBS pH 7.4 (in presence of 3% DMSO).

Supplementary Table 6. Summary of the fluorescence lifetimes of the peptides **SP1A**, **SP1B**, **LP1A**, **LP1B,** and the mixtures (**SP1A**+**LP1B**) and (**SP1B**+**LP1A**). All the peptides were excited at λ_{ex}= 290nm; Concentration of all the peptides wassame = 20µM; k_f and k_{nr} are weighted means from the biexponential fits: $k_f = \phi/\tau$ and $k_{nr} = (1-\phi)/\tau$.[9]

**Peptide**	$\boldsymbol{\phi_f}$	**λ_{em}(nm)**	**τ (ns)**	**k_f**	**k_{nr}**	χ^2
**SP1A**	0.076	355	2.07	0.037	0.446	1.07
**LP1B**	0.021	495	4.06	0.005	0.241	1.21
**(SP1A + LP1B)**	0.059	355	1.47	0.040	0.640	1.06
	0.059	495	4.37	0.014	0.215	1.12
**LP1A**	0.241	355	1.90	0.126	0.399	1.01
**SP1B**	0.081	495	5.21	0.015	0.176	1.66
**(SP1B + LP1A)**	0.245	355	0.74	0.331	1.02	1.09
	0.245	495	7.23	0.033	0.104	1.13

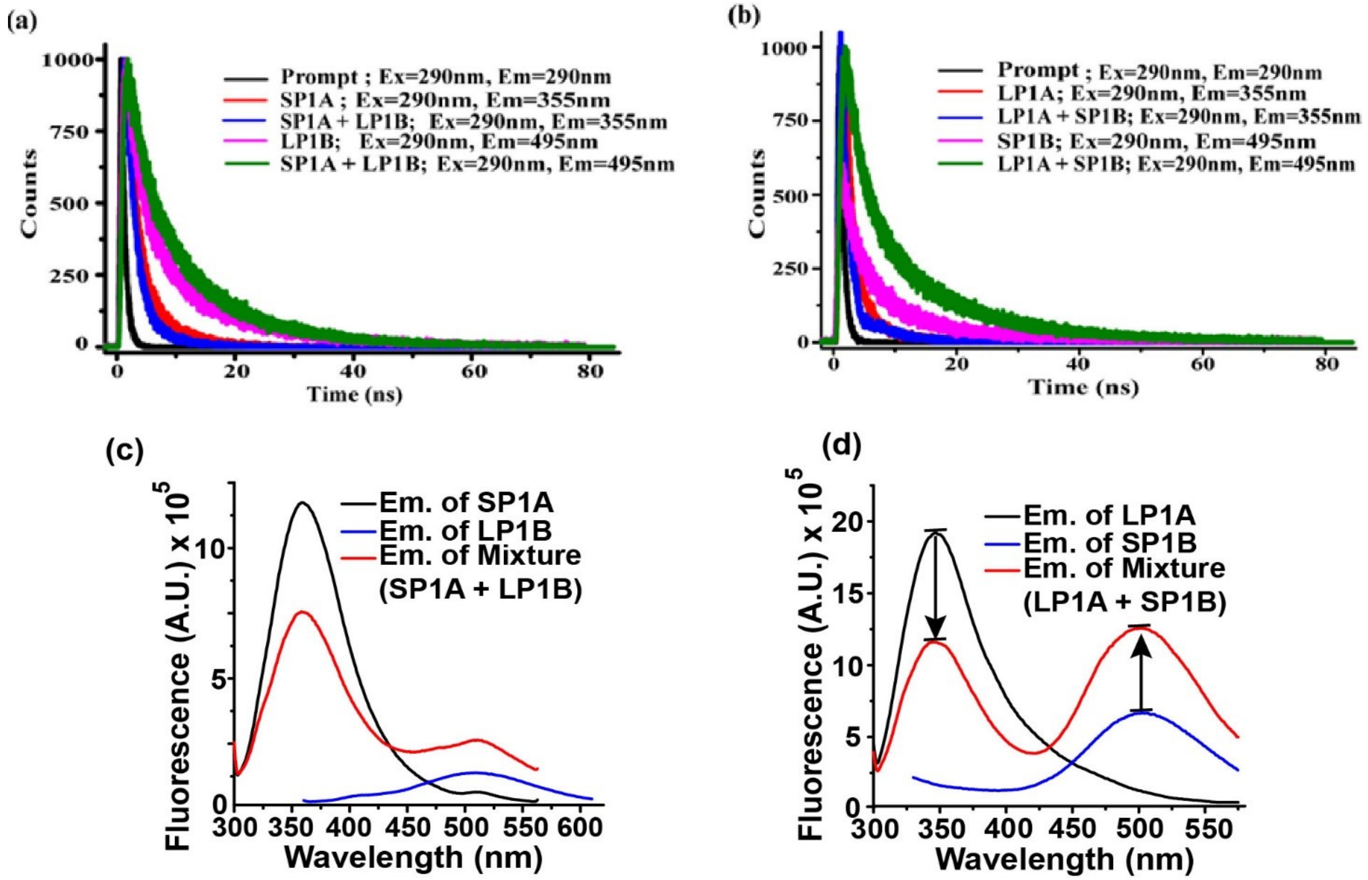

Supplementary Figure19. (a) Time-resolved fluorescence spectra of the peptides **SP1A** (Red, Ex=290nm, Em=355nm), **SP1A+LP1B** (Blue, Ex=290nm, Em=355nm), **LP1B** (Purple, Ex=290nm, Em=495nm) and **SP1A+LP1B** (Green, Ex=290nm, Em=495nm). (b) Time-resolved fluorescence spectra of the peptides **LP1A** (Red, Ex=290nm, Em=355nm), **LP1A + SP1B** (Blue, Ex=290nm, Em=355nm), **SP1B** (Purple, Ex=290nm, Em=495nm) and **LP1A+SP1B** (Green, Ex=290nm, Em=495nm). Spectra were recorded in 20μM of peptide solutions in PBS pH 7.4 (in the presence of 3% DMSO). c. Emission spectra of **SP1A** (Black, Ex=290nm), **LP1B** (Blue, Ex=320 nm), **SP1A+LP1B** (Red, Ex=290 nm). **d.** Emission spectra of **LP1A** (Black, Ex=290 nm), **SP1B** (Blue, Ex=320 nm), **LP1A+SP1B** (Red, Ex= 290 nm).

Supplementary Table 7. Summary of the fluorescence lifetimes of the **SP1A**, **SP1B**, **SP1C,**and the mixture (**SP1A**+**SP1B**) in the presence of $A\beta_{40}$. All the peptides were excited at λ_{ex} = 290 nm; Concentration of all the peptides were same = 20μM; $<\tau>$, k_f and k_{nr} are weighted means from the biexponential fits: $<\tau> = 1/ (\alpha_1/\tau_1 + \alpha_2/\tau_2)$, $k_f = \Phi/<\tau>$, and $k_{nr} = (1-\Phi_f)/<\tau>$.[9]

**peptide**	$\boldsymbol{\Phi_f}$	$\boldsymbol{\lambda_{em}}$ **(nm)**	$\boldsymbol{\tau_1}$ **(ns)**	$\boldsymbol{\tau_2}$ **(ns)**	$\boldsymbol{<\tau>}$ **(ns)**	$\mathbf{k_f}$ **($\mathbf{10^8\ s^{-1}}$)**	$\mathbf{k_{nr}}$ **($\mathbf{10^8\ s^{-1}}$)**	$\boldsymbol{\chi^2}$
**SP1A**	0.075	355	1.261 (34%)	3.256 (66%)	2.577	0.29	3.591	0.99
**SP1B**	0.039	495	3.223 (26%)	16.985 (74%)	13.367	0.029	0.719	1.05
**SP1C**	0.26	355	1.098 (75%)	3.313 (25%)	1.636	1.589	4.522	0.99
	0.26	495	6.090 (15%)	16.519 (85%)	14.968	0.174	0.494	1.02
Mixture peptide (**SP1A+SP1B**)	0.114	355	1.548 (43%)	3.421 (57%)	2.615	0.434	3.389	1.00
	0.114	495	3.430 (27%)	16.60 (73%)	13.090	0.087	0.677	1.04

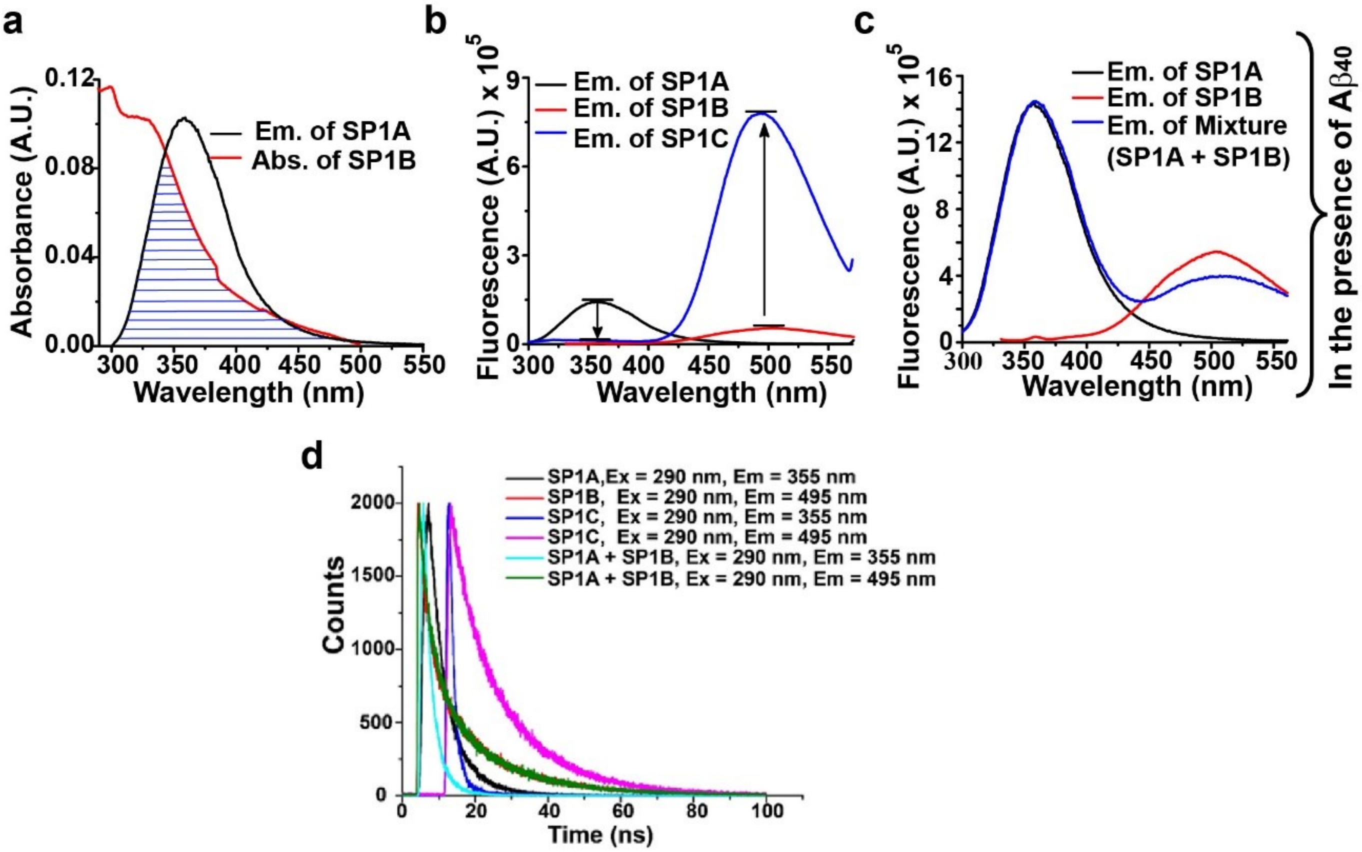

Supplementary Figure20. a. Overlap of the emission spectrum of the donor, **SP1A** (black), and the absorbance spectrum of the acceptor, **SP1B** (red), in the presence of $A\beta_{40}$. **b.** Emission spectra of **SP1A** (black), **SP1B** (red), and **SP1C** (blue) in the presence of $A\beta_{40}$. **c.** Emission spectra of **SP1A** (black), **SP1B** (red), and the mixture, **SP1C+SP1B** (blue), in the presence of $A\beta_{40}$. Spectra were recorded with 20 µM solutions of the peptides in PBS (50 mM) pH 7.4.**d.**Time resolved fluorescence spectra of the peptide **SP1A** (black, λ_{ex} = 290 nm, λ_{em} = 355 nm), **SP1B** (red, λ_{ex} = 290 nm, λ_{em} = 495 nm), **SP1C** (for blue, λ_{ex} = 290 nm, λ_{em} = 355 nm and for magenta, λ_{ex} = 290 nm, λ_{em} = 495 nm) and mixture, **SP1A**+**SP1B** (for cyan, λ_{ex} = 290 nm, λ_{em} = 355 nm and for olive, λ_{ex} = 290 nm, λ_{em} = 495 nm) in presence of $A\beta_{1-40}$. Spectra were recorded in 20µM of peptide solutions in PBS pH 7.4 (in presence of 3% DMSO).

4. References

1 G. Malgieri, G. D'Abrosca, L. Pirone, A. Toto, M. Palmieri, L. Russo, M. F. M. Sciacca, R. Tatè, V. Sivo, I. Baglivo, R. Majewska, M. Coletta, P. V. Pedone, C. Isernia, M. De Stefano, S. Gianni, E. M. Pedone, D. Milardi and R. Fattorusso, *Chem. Sci.*, 2018, **9**, 3290–3298.

2 M. Konar, S. Bag, P. Roy and S. Dasgupta, *Int. J. Biol. Macromol.*, 2017, **103**, 1224–1231.

3 J. McLaurin and A. Chakrabartty, *J. Biol. Chem.*, 1996, **271**, 26482–26489.

4 M. Traïkia, D. E. Warschawski, M. Recouvreur, J. Cartaud and P. F. Devaux, *Eur. Biophys. J.*, 2000, **29**, 184–195.

5 J. R. Lakowicz, in *Principles of Fluorescence Spectroscopy*, Springer US, Boston, MA, 2006, pp. 443–475.

6 J. R. Lakowicz, *Principles of fluorescence spectroscopy*, Springer, 2006.

7 T. Sakurai, T. Iwasaki, T. Okuno, Y. Kawata and N. Kise, *Chem. Commun.*, 2011, **47**, 4709.

8 C. Berney and G. Danuser, *Biophys. J.*, 2003, **84**, 3992–4010.

9 S. S. Bag, S. Jana, A. Yashmeen, K. Senthilkumar and R. Bag, *Chem. Commun.*, 2014, **50**, 433–435.

10 T. Mosmann, *J. Immunol. Methods*, 1983, **65**, 55–63.

11 C. P. LeBel, H. Ischiropoulos and S. C. Bondy, *Chem. Res. Toxicol.*, 1992, **5**, 227–231.

12 M. Martínez, N. Martínez and W. Silva, *Bio-protocol*, 2017, **7**, e2411.

13 C. A. Paul, B. S. Beltz and J. Berger-Sweeney, *Discovering neurons : the experimental basis of neuroscience*, Cold Spring Harbor Laboratory Press, 1997.

14 J. Buttner-Ennever, *J. Anat.*, 1997, **191**, 315–317.

15 G. Paxinos and C. Watson, *Elsevier Acad. Press*, 2007, **170**, 547–612.
